# Quantitative Online Monitoring of an Immobilized Enzymatic Network by Ion Mobility–Mass Spectrometry

**DOI:** 10.1021/jacs.4c04218

**Published:** 2024-07-16

**Authors:** Quentin Duez, Jeroen van de Wiel, Bob van Sluijs, Souvik Ghosh, Mathieu G. Baltussen, Max T. G. M. Derks, Jana Roithová, Wilhelm T. S. Huck

**Affiliations:** Institute for Molecules and Materials, Radboud University, Heyendaalseweg 135, Nijmegen 6525 AJ, The Netherlands

## Abstract

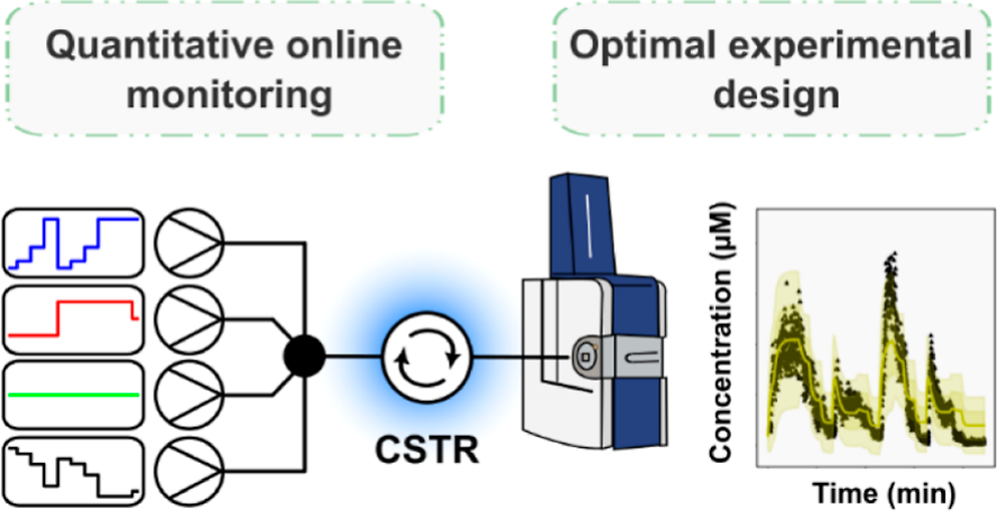

The forward design of in vitro enzymatic reaction networks (ERNs) requires a detailed analysis of network kinetics and potentially hidden interactions between the substrates and enzymes. Although flow chemistry allows for a systematic exploration of how the networks adapt to continuously changing conditions, the analysis of the reaction products is often a bottleneck. Here, we report on the interface between a continuous stirred-tank reactor, in which an immobilized enzymatic network made of 12 enzymes is compartmentalized, and an ion mobility–mass spectrometer. Feeding uniformly ^13^C-labeled inputs to the enzymatic network generates all isotopically labeled reaction intermediates and products, which are individually detected by ion mobility–mass spectrometry (IMS–MS) based on their mass-to-charge ratios and inverse ion mobilities. The metabolic flux can be continuously and quantitatively monitored by diluting the ERN output with nonlabeled standards of known concentrations. The real-time quantitative data obtained by IMS–MS are then harnessed to train a model of network kinetics, which proves sufficiently predictive to control the ERN output after a single optimally designed experiment. The high resolution of the time-course data provided by this approach is an important stepping stone to design and control sizable and intricate ERNs.

## Introduction

Living systems exploit complex cascades of enzymatic reactions to achieve key functions such as energy metabolism or maintaining homeostasis in changing environments.^1,2^ Taking inspiration from biological systems, significant progress has recently been made in the forward design of in vitro enzymatic reaction networks (ERNs) with specific functionalities, ranging from the synthesis of added-value chemicals to the recycling of cofactors or processing of molecular inputs according to logic-gate responses.^3−11^ The design of increasingly sizable and complex ERNs introduces crosstalk such as substrate competition, allostery, or inhibition. Achieving control and optimization toward desired outcomes, such as minimizing side products or limiting cofactor consumption, necessitates a thorough understanding of the kinetic parameters and interactions within ERNs, including those that are not readily identifiable.^12−17^

Flow chemistry emerges as particularly beneficial for determining network kinetics, facilitating the systematic exploration of a large input space while analyzing the resulting mixture of products.^16,18^ In a previous work, we streamlined this search with an optimal experimental design (OED) workflow,^19^ utilizing an OED algorithm to design maximally informative inflow profiles of metabolites into a flow reactor.^20−22^ With this tool, we were able to train a model that could reliably control the nucleotide salvage pathway immobilized on beads, comprising 6 enzymes.^19^

Although OED workflows eliminate the need to systematically probe individual reactions, the analysis of ERN products constitutes a bottleneck when it is carried out offline using chromatographic methods.^13,23^ In our previous work, the offline analysis of ERN products by HPLC resulted in relatively low sampling rates (3–9 min per sample) and limited observability over intermediate metabolites. Because of this, several design-build-test iterations were required to train a model that could control the reactions in the network.^19^ To facilitate the optimization of an ERN, it is imperative to employ measurement techniques that fulfill three essential requirements: (i) many intermediates need to be observed quantitatively, including low-concentration species; (ii) the time resolution of the observations needs to be significantly faster than the input changes suggested by the OED algorithm; and (iii) the approach needs to be time- and cost-effective.

High-throughput approaches have been developed to monitor biocatalytic transformations in real time; among them are implementations of benchtop NMR and/or IR/UV spectrometers coupled to flow reactors.^24−26^ Yet, probing multistep reaction cascades often requires sophisticated analysis to resolve, assign, and quantify overlapping signals.^24,27,28^ Moreover, the sensitivity of currently reported NMR approaches precludes the detection of low-concentration species, meaning that these approaches fall short of our requirements.

The direct coupling between flow reactors and electrospray ionization–mass spectrometry (ESI–MS) alleviates some of these issues.^12,16,29−35^ MS overcomes the difficulties associated with the analysis of complex reaction mixtures because individual compounds are detected based on their mass-to-charge ratios. Moreover, interfacing ESI–MS with ion mobility spectrometry (IMS) enables the separation of isomeric ions in the gas phase based on their size and shape.^36^ Quantitative MS can be achieved by comparing ion intensities with pre-established calibration curves.^12,33,35^ However, the comparison between ion intensities and concentrations in solution is jeopardized by matrix effects arising from the analysis of crude reaction mixtures, which can affect the ionization efficiency of the compounds of interest.^37^ Instead, because isotopologues share the same ionization efficiency regardless of matrix effects, their relative intensities in a mass spectrum directly relate to their relative concentrations in solution.^38,39^ By diluting the crude output from an ERN with isotopically labeled standards of known concentrations, it becomes possible to quantify the analytes of interest by ESI–MS, regardless of matrix effects. As shown in the pioneering works of Panke and co-workers, such an approach has been employed to quantify product mixtures and determine reaction parameters for ERN subsystems composed of up to four enzymes added sequentially to the reaction medium through a series of experiments involving systematic variations in enzyme/substrate addition sequences.^16^

In this work, we elaborate on the analytical techniques and demonstrate that it is possible to train a model that maps the dynamics of the entire glycolysis network in flow within a single design-build-test cycle of the OED workflow. We quantitatively monitor ERN dynamics by feeding a uniformly ^13^C-labeled substrate to a network comprising 12 enzymes of the glycolysis pathway. The generation of isotopically ^13^C-labeled intermediates in situ enables their quantification using available, nonisotopically labeled standards. We used a similar flow chemistry platform described before, in which we immobilized all enzymes constituting the network on hydrogel beads for compartmentalization in a filtered continuous stirred-tank reactor (CSTR).^18,19^ Confining enzymatic reactivity to the CSTR prevents the loss of enzymes and allows us to disregard the downstream reactivity after metabolites leave the reactor. The network output was monitored in real time under continuously changing reactor conditions, enabling the collection of quantitative data for individual metabolites at a 550 ms time resolution (∼100,000 data points per experiment). Moreover, we included IMS separation to detect, identify, and quantify isomeric metabolites.^40^ By fulfilling the three essential analytical requirements identified above, we drastically simplified the procedure to gain control over ERNs.

## Results and Discussion

### Interfacing the Compartmentalized Glycolysis Pathway with ESI–IMS–MS for Online Quantitative Monitoring

**Figure 1:**
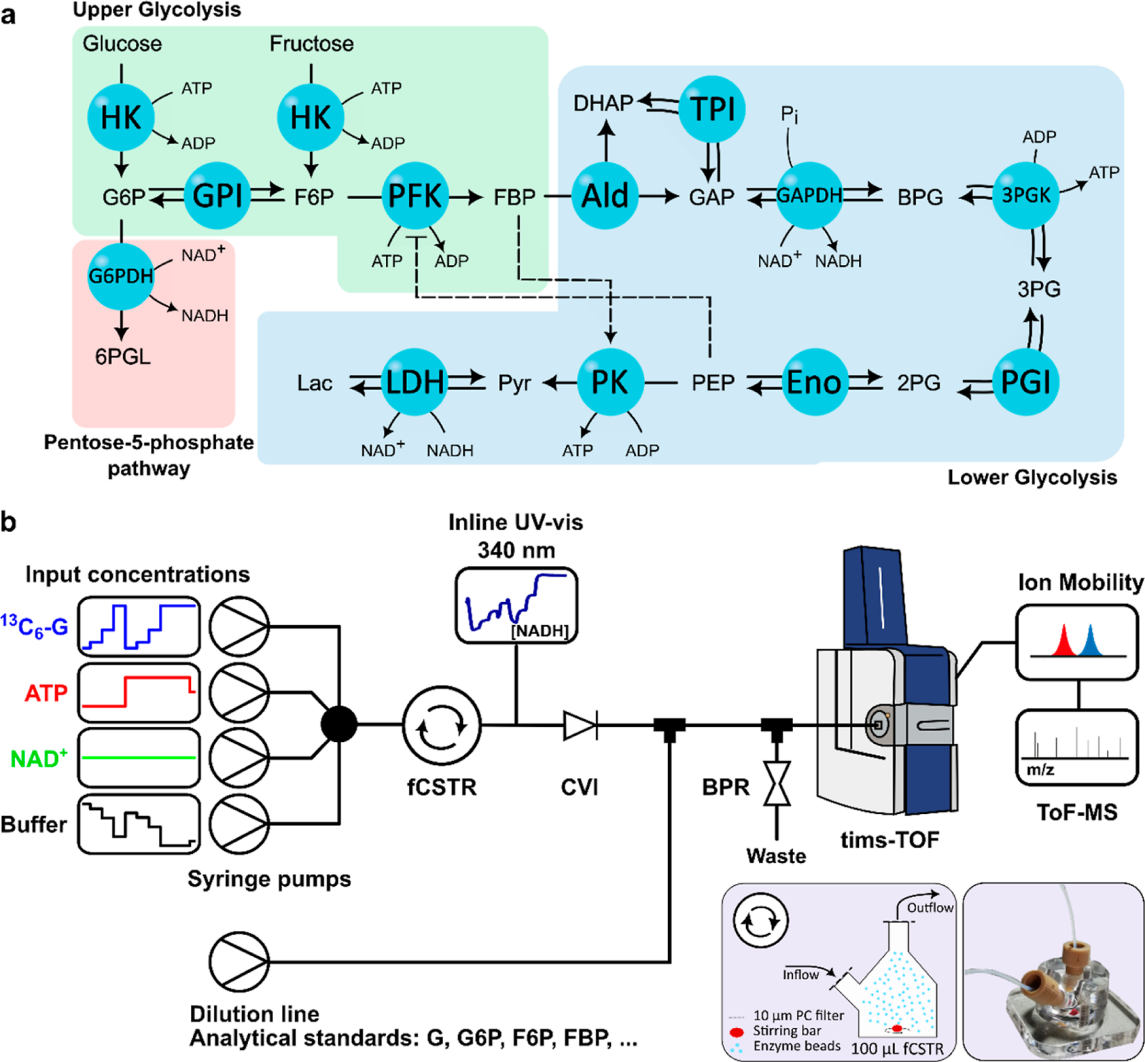
(a) Enzymatic network investigated in this work. Each enzyme is immobilized on hydrogel beads, represented by blue spheres. (b) Schematic representation of the interface between a CSTR, in which the ERN is compartmentalized, and an ion mobility–mass spectrometer. Reaction conditions are controlled by the flow rates of syringe pumps, and the outflow of the CSTR is analyzed by inline UV spectroscopy. The flow crosses a check valve inlet and is diluted by a mixture of isotopologues with a known concentration. The total flow is then diverted between the mass spectrometer and a waste line. The total pressure in the system is controlled by a BPR.

The in vitro ERN selected in this work is composed of glycolytic enzymes and can be subdivided into two parts: The “upper” part consumes two equivalents of ATP to convert a hexose into fructose-1,6-biphosphate (FBP), and the “lower” part converts the FBP to two equivalents of pyruvate (Pyr), each yielding 2 equiv of ATP. Allosteric regulation plays an important role in mediating the activity between these two parts: FBP is an allosteric activator of PK, whereas phosphoenolpyruvate (PEP) is an allosteric inhibitor of PFK (Figure 1a). In total, the ERN comprises a total of 13 reactions catalyzed by 12 enzymes in a single reactor. We also included G6PDH from the pentose phosphate pathway to introduce substrate competition between GPI and G6PDH and to mediate the concentration of NADH from which Pyr can be reduced to lactate (Lac).

To compartmentalize the ERN in a flow reactor, we individually immobilized each enzyme on hydrogel beads^10,18^ via the coupling between lysine residues and NHS-activated carboxylic acids on the hydrogel beads (see Supporting Information—Sections S2 and S3). Selected volumes (Tables S5–S9) of functionalized beads were pipetted into a CSTR (equipped with a filter on both the inlet and outlet to prevent the escape of the beads), to which we continuously flow the reaction inputs using programmable syringe pumps. The average residence time in the CSTR is defined by the total flow rate, and reaction conditions are controlled by the individual inflows of reagents (a ^13^C_6_-hexose and cofactors ATP and NAD^+^—Tables S5–S9).

**Figure 2:**
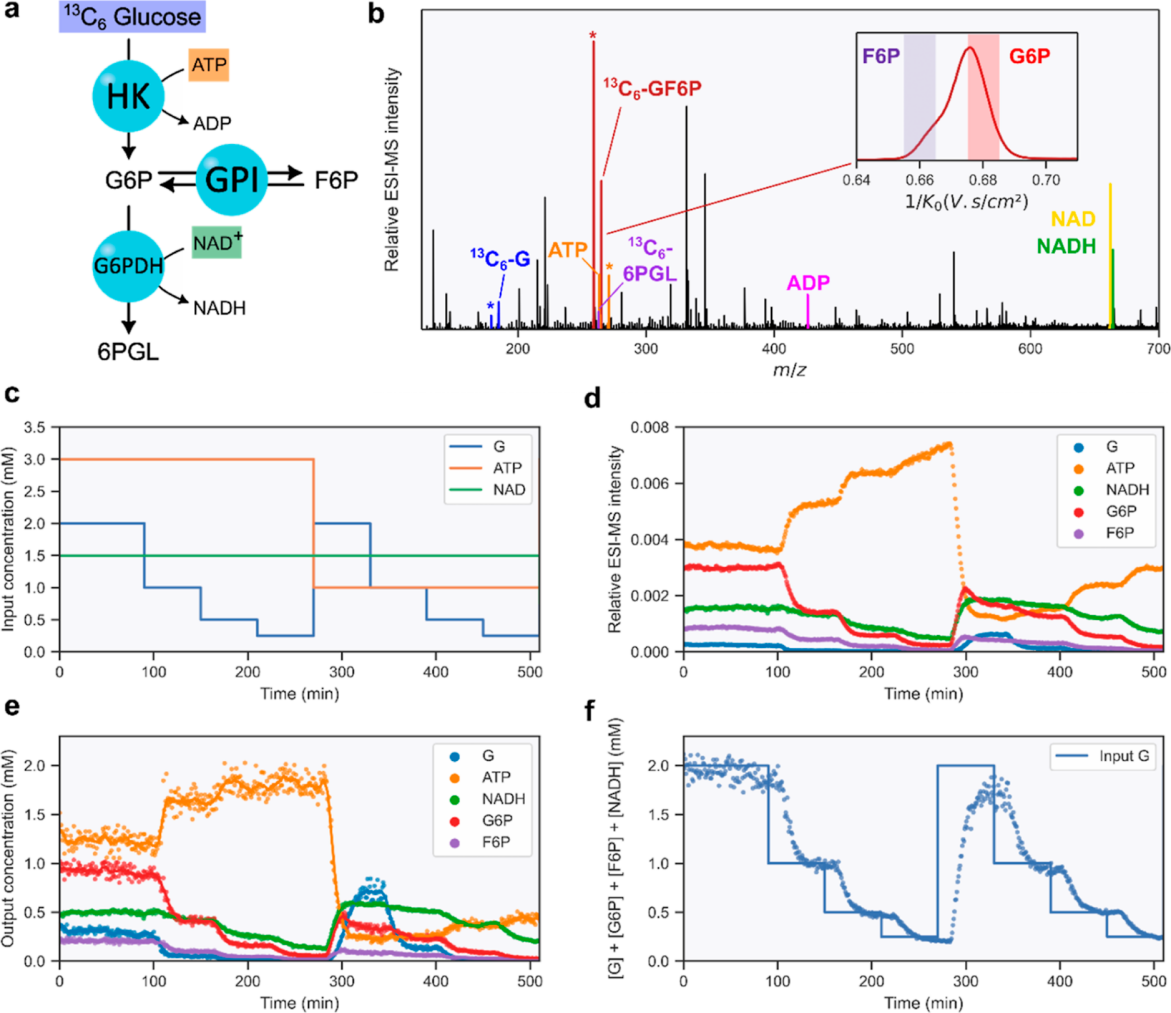
(a) Subsystem of the upper glycolysis composed of HK, GPI, and G6PDH, compartmentalized in the CSTR. (b) ESI–MS analysis of the outflow of the CSTR. The output products from the ERN are highlighted with colored bars, and the standard compounds used for quantification are highlighted by asterisks (*). Inset: ion mobility separation of the isomers G6P and F6P (^13^C_6_-GF6P, *m*/*z* 265.04). (c) Modulations of input concentrations to the ERN. As described in Supporting Information, the flow profile is preceded by 2 h of equilibration time. (d) Time evolution of ion intensities resulting from the input modulations. (e) Output concentrations measured in real time by ESI–MS. Dots correspond to binned data, and lines correspond to rolling averages (*n* = 10). (f) Comparison between the summed concentrations of substrate and product metabolites (G, G6P, F6P, and NADH as a proxy for 6PGL) and the input concentration of G.

We first examined a cascade of reactions catalyzed by a subsystem of 3 enzymes from the upper glycolysis (Figure 2a). This network consists of HK, GPI, and G6PDH and converts glucose (G) into 6-phosphogluconolactone (6PGL) and fructose-6-phosphate (F6P) through glucose-6-phosphate (G6P). We fed a controlled input of ^13^C_6_-labeled G, ATP, and NAD^+^ to the CSTR and continuously monitored the product mixture by ESI(−)–MS. Individual metabolites were detected and identified based on their mass-to-charge ratios. All expected intermediates, products, and cofactors were observed as [M – H]^−^, except for ATP, which was detected as [M – 3H + Na]^2–^ (Figure 2b and Table S1). Although G6P and F6P cannot be resolved by MS alone, they are readily separated by IMS based on their size and shape. As shown in Figure 2b, F6P appears at a lower inverse mobility (1/*K*_0_) than G6P, in agreement with previously reported collision cross-section values.^40^ The abundance of each isomer can be extracted individually for selected inverse mobility ranges (Figure S1).

We then systematically varied the input concentrations of ^13^C_6_-glucose and ATP in steps and continuously monitored the metabolic flux of the ERN under changing conditions. Mass spectra are collected every 550 ms, which corresponds to more than 6000 data points per input concentration. To reduce the noise, the raw data were binned at 45 s intervals. As shown in Figure 2c,d, the relative abundance of all cofactors and ^13^C-labeled metabolites produced by the ERN evolved with the input modulations.

The quantification of metabolites was achieved by analyzing the outflow of the CSTR by inline UV spectroscopy and diluting with nonisotopically labeled standards before infusion into the ESI source of our IMS–MS instrument (Figure 1b). Feeding a uniformly ^13^C-labeled substrate to the ERN yielded ^13^C-labeled intermediates and products in situ, which were quantified by comparing their ESI–MS ion intensities with the intensities of nonisotopically labeled standards of known concentrations (see Supporting Information—Sections S7 and S8). To quantify the cofactor output, we used (i) an isotopically labeled ATP standard and (ii) inline UV absorbance measurements at a fixed wavelength. The UV absorbance data were used to establish a calibration that allows for the determination of NADH concentrations from ion intensities (Supporting Information—Section S10). Because isotopically labeled standards were not available to us, the quantification of NAD^+^ and ADP was left out. For the subsystem shown in Figure 2, quantification was achieved for ATP, NADH, and three of the ^13^C-labeled metabolites (Figure 2e). The relative intensities of the other metabolites (6PGL, ADP, and NAD^+^) are shown in Figure S2.

Because of the stoichiometry of the reaction catalyzed by G6PDH, the concentration of 6PGL can be inferred from the measured concentrations of NADH. Gratifyingly, summing the measured concentrations of all ^13^C-labeled metabolites G, G6P, F6P, and NADH as a proxy for 6PGL correlates well with the input feed of ^13^C_6_-glucose (Figure 2f). The delay observed between input modulations and the response of the ERN corresponds to the time needed to refresh and detect the contents of the CSTR. As shown with this small subnetwork, the quantitative online monitoring of the reactor output yields real-time information about the ERN response in changing environments.

### Quantitative Online Monitoring of the Glycolytic ERN

We then moved on to the analysis of the entire ERN, which consists of 13 reactions catalyzed by 12 enzymes (Figure 1a). The separate analysis of individual metabolites produced in the ERN revealed that some of them undergo fragmentation either in the ionization source or in the ion optics of the mass spectrometer. Ion fragmentation can be an issue for quantification when the ion fragments are detected at the same *m*/*z* as the metabolites of interest. For instance, the ESI–MS analysis of a fructose 1,6-biphosphate (FBP) standard shows the presence of F6P in the mass spectrum, resulting from the loss of neutral HPO_3_ (Figure S25). The contribution of FBP fragmentation to the ion intensity of F6P could lead to an overestimation of the F6P concentration in our experiments. Assuming that (i) isotopologues fragment with similar rates and that (ii) the fragmentation rate remains constant throughout the experiments, the analysis of metabolite standards under the same instrumental conditions as our experiments provides estimates of the fragmentation rate for each metabolite. The contribution of in-flight fragmentation can then be subtracted for quantification experiments (see Supporting Information—Section S9).

**Figure 3:**
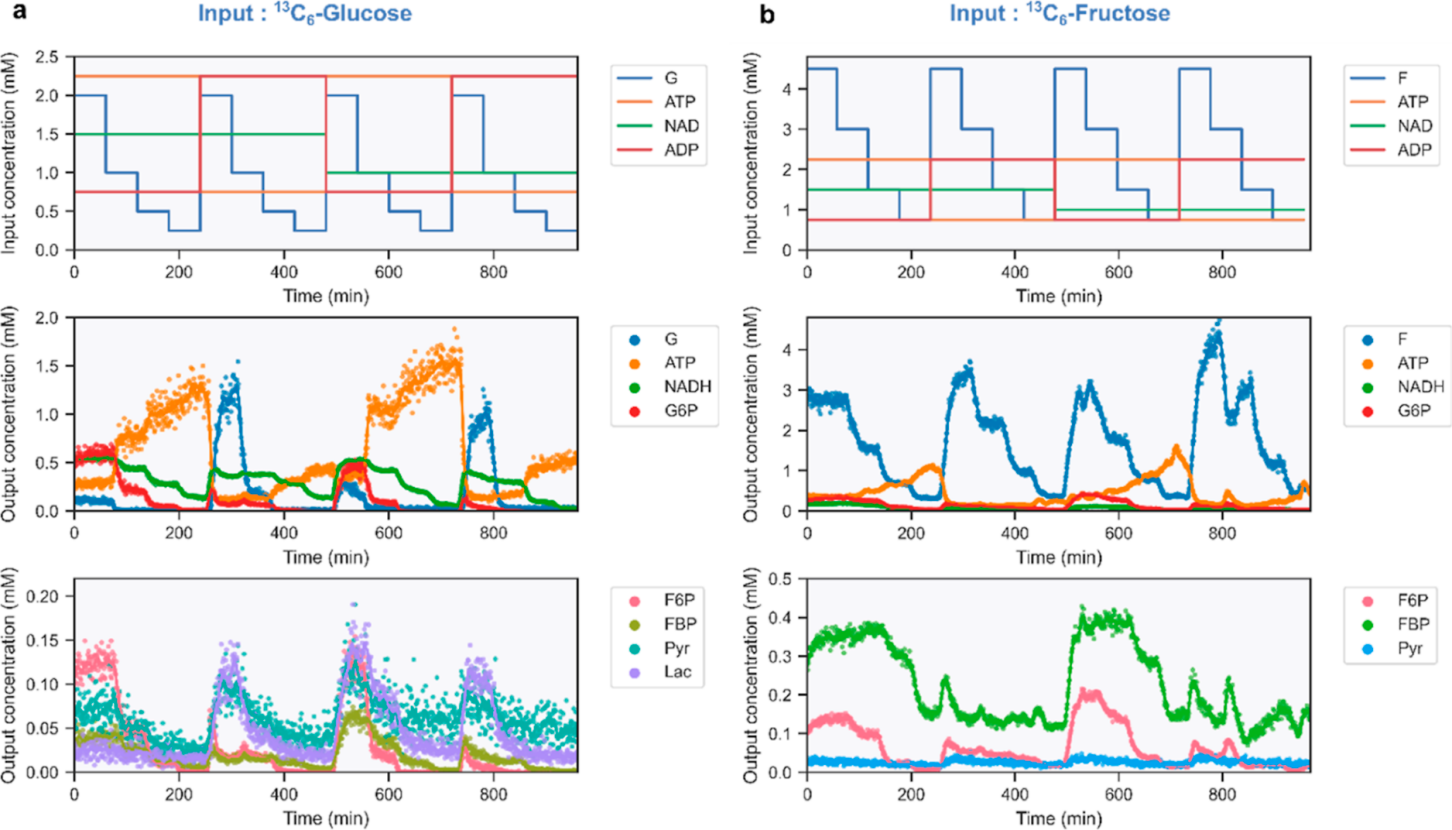
(a) Input modulations for the glycolytic ERN (Figure 1a), starting from ^13^C-glucose and resulting output concentrations. (b) Input modulations for the glycolytic ERN, starting from ^13^C-fructose and the resulting output concentrations. Dots correspond to binned data, and lines correspond to rolling averages (*n* = 10).

The experimental design for monitoring the entire ERN is similar to that described in the previous paragraph. However, we now also include a variable input concentration of ADP as it is a cofactor for 3PGK and PK. By using either ^13^C_6_-glucose or ^13^C_6_-fructose as input substrates, we observed 13 metabolites of glycolysis (Table S1) and were able to quantify eight of them (Figure 3). The relative intensities of the metabolites that we could not quantify are shown in Figures S3 and S4.

The ions corresponding to BPG, 2/3PG, and PEP were not detected, probably because of their low concentrations in solution. The end-products of the glycolytic network, Pyr and Lac, are still observed because they do not react further. To estimate the lower bound of metabolite concentrations we can determine in our experiments, we determined the concentration that would be associated with the intensity of the background noise at the *m*/*z* of the expected 2/3PG and PEP ions (Figure S5). We estimate this lower limit to be ∼20 μM, which is consistent with the output concentrations determined for the observable metabolites. In the experiment starting with ^13^C_6_-fructose as the input substrate (Figure 3b), the concentration of Lac is too close to this value to be accurately determined.

The comparison of both hexose substrates reveals differences regarding their incorporation in the network. The nature of the hexose substrate modifies the entry point in the glycolytic network. The fructose input is being phosphorylated by HK directly into F6P, bypassing the isomerization step carried out by GPI (Figure 1a). The fructose entry point thus yields a higher concentration of FBP. The lower Lac generation may be attributable to the lower NADH concentration compared to the G experiment as GPI serves as a bottleneck for 6PGL generation. As observed in Figure S6, the relative conversion of ^13^C_6_-fructose into other metabolites is significantly lower than for ^13^C_6_-glucose. This difference can be attributed to a lower affinity of HK for d-fructose than that for d-glucose.^41^

Summing the concentrations of the observable metabolites also reveals differences between both hexoses (Figure S7). For the ^13^C_6_-fructose experiment, summing the output concentrations of fructose, G6P, F6P, FBP, and Pyr correlates well with the input feed of ^13^C_6_-fructose. Conversely, the summed output concentrations of observable metabolites do not match the input feed of ^13^C_6_-glucose. This indicates that the concentration of 6PGL or XAP (DHAP/GAP), which we observe but cannot quantify, is higher when using G as a substrate, especially at high ATP concentrations. As mentioned above, BPG, 2/3PG, and PEP are not detected. Finally, we evaluated the repeatability of our approach by reproducing the experiment shown in Figure 3b with a new mixture of enzyme beads and with different stock solutions. Figure S8 shows the robustness of our approach as it yields consistent output concentrations across measurement days, even for lower concentration ranges.

### Harnessing MS Data and OED to Train a Kinetic Model of the Glycolysis Pathway

The quantitative online monitoring approach described in this work nicely complements the active learning workflow we recently reported.^19,42^ Therefore, we assessed whether the increased information contained in the IMS–MS data would yield a trained model within a single design-build-test cycle for the entire ERN. Compared to previous works, this would drastically simplify the procedure to gain full control over the output of ERNs (see eqs S5–S8).^19,42^

**Figure 4:**
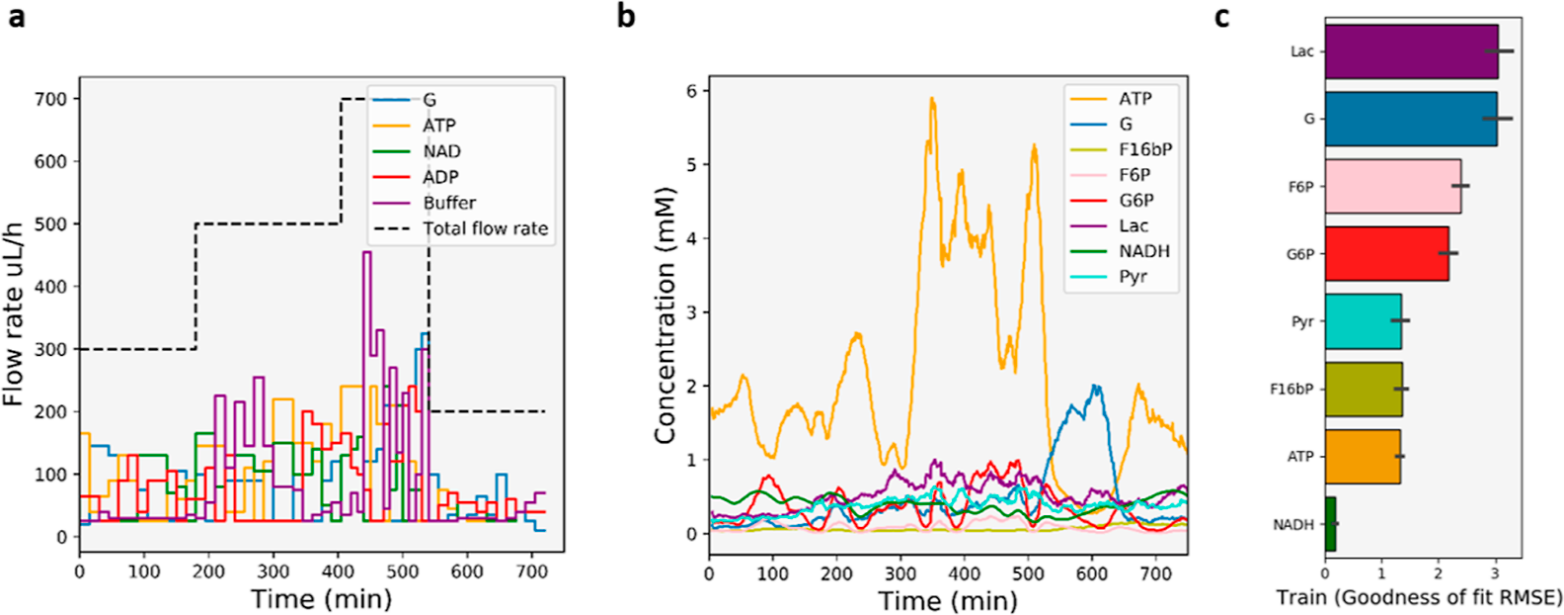
Input concentrations and output data used to train a model of the glycolysis pathway. (a) Optimally designed input profile of the different substrates; the black dashed line represents the total flow rate over time, indicating 4 residence time regimes. Every 15 min, the input flow rates are varied. (b) Resulting output concentrations for 8 species, in order: ATP, G, FBP, F6P, G6P, Lac, NADH, and Pyr; the lines represent rolling averages (c) Goodness-of-fit score of the fit of the model to the training data; the values on the *x*-axis represent the RSME of the fit to the raw data.

First, we defined a model of ordinary differential equations for the glycolysis pathway (56 kinetic parameters) and used the OED algorithm to design a pulse experiment for the reaction inputs (see eqs S4–S8). Figure 4a shows the corresponding pulse sequence of the four inputs: ATP, G, NAD, and ADP; each species is marked by their respective inflow rates into the reactor. Figure 4b subsequently shows the corresponding time course data monitored by IMS–MS for the 8 observable species (G, G6P, F6P, FBP, ATP, NADH, Lac, and Pyr). After training the model using this time course data set (Figure 4c), we needed to test if it could predict conditions outside those used to train it. Therefore, we simulated the outcome of the experiment shown in Figure 3a by using the newly trained model. We specifically chose this experiment because it was carried out in a reactor with different enzyme concentrations and different stock concentrations of metabolites within the syringes, thus ensuring a larger divergence between the test and training data (enzyme and stock concentrations in Tables S6 and S8).

**Figure 5:**
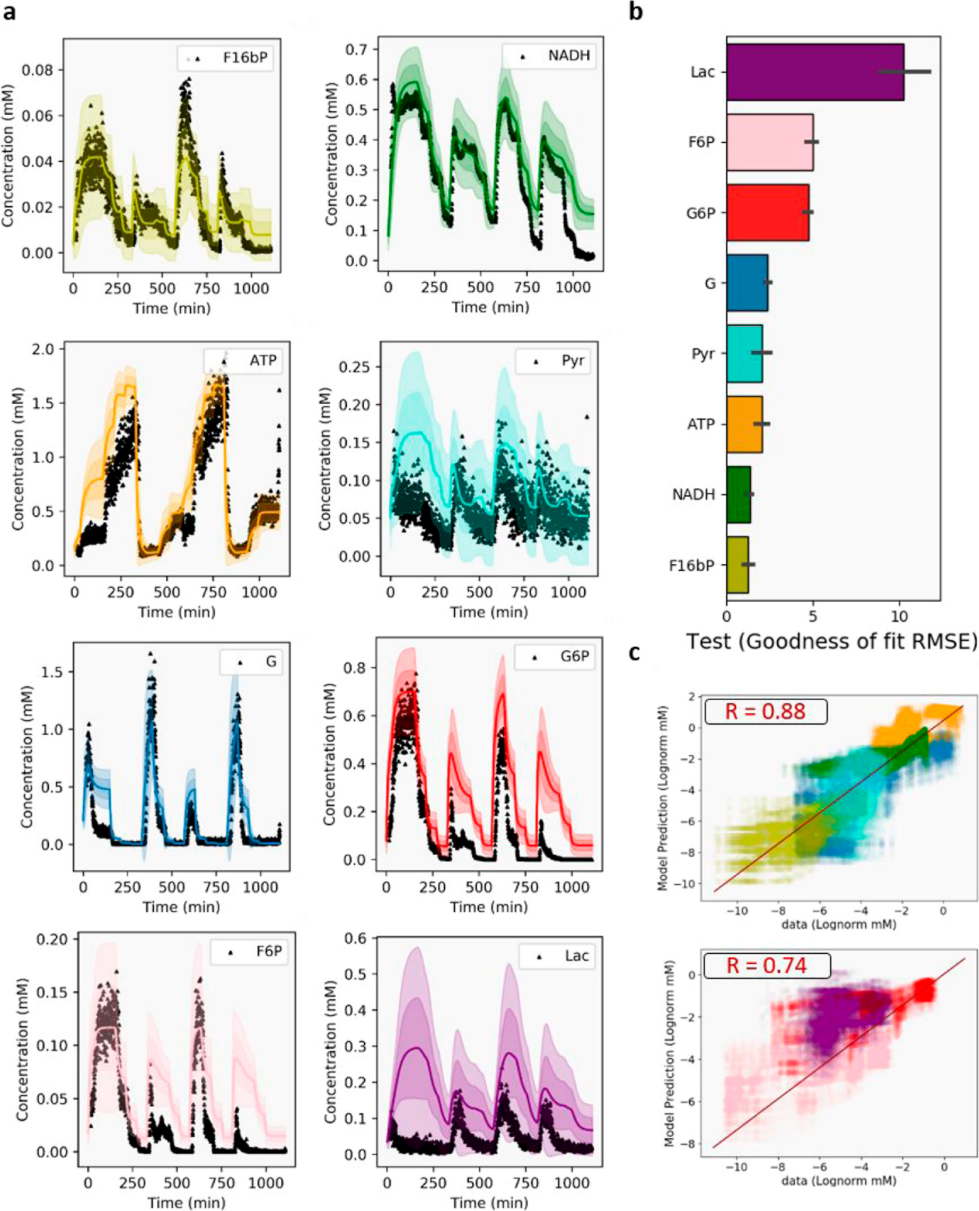
Overview of the model’s prediction of the test data. Both stock concentrations within syringes and enzyme concentrations differ between the test and training data. (a) Prediction of the experiment shown in Figure 3a by the newly trained model (*N* = 100) after training it on a single OED data set. The line shows the mean and the different shades of the prediction within one (darker shade) and two (lighter shade) standard deviations, respectively. (b) Goodness-of-fit score for the model predicting the test data. The values on the *x*-axis represent the RSME of the prediction to the data. The order of the species in the bar plot unsurprisingly resembles the goodness of fit for the training data. (c) Regression plot for the concentration of the species in the data (*x*-axis) and the prediction by the model (*y*-axis). Breaking the RMSE values down with an *R*-value for the 5 species that are very accurate including G, Pyr, ATP, NADH, and FBP (*R* = 0.88, top) and the three species that deviate more including Lac, F6P, and G6P (*R* = 0.74, right). The comparison includes simulation for the fittest 10 parameter sets, and the depth of color reflects the frequency of individual data points.

The results in Figure 5 summarize the predictive power of the model after a single OED experiment. First, it shows that the predictions are quantitatively accurate for the majority of the quantified metabolites across 16 different input conditions. Second, the remaining uncertainty for these predictions is manageable, as evidenced by the standard deviations marked as shaded areas (Figure 5a). Finally, the metabolites we did not detect (2/3PG, PEP, and BPG) are predicted to be present with concentrations in the medium lower than the bounds of detection that we estimated (Figure S10).

We note some divergence in the root mean squared error (RSME) score between Lac, G6P, and F6P and the other species (Figure 5b). As shown in Supporting Information, we observed that an isomer of Lac originates from in-flight fragmentation of G (Figure S27). The output concentration of Lac is thus determined under the assumption of a consistent fragmentation rate across experiments (see the previous paragraph and Supporting Information—Section S9). Because Lac is a consistent outlier for our model, this assumption is probably not accurate. Accordingly, the model could not approximate Lac concentrations when using test data as training data (Figure S9). For G6P and F6P, the lower ATP concentration regime is slightly overestimated compared to other metabolites (second and fourth series of steps—Figure 5c). This is not surprising since the goodness-of-fit scores of the model to the training data show that these species were among those which deviated most (Figure 4c), indicating that these lower concentration regimes were not adequately mapped by the model and the OED experiment. Regardless, considering that a single OED iteration was previously not sufficient to even approximate the data quantitatively,^19^ the predicted concentrations from Figure 5 are remarkably close to the experimental data.

In Figure S11, we underscore this by quantifying in silico the efficiency of utilizing our online monitoring approach. We show that the estimated gain in information about the kinetics per minute of experimental time is higher than that of other types of experimental setups. Compared to previous reports,^16,39^ this proof of principle shows that harnessing highly informative observations enhances our capacity to control in vitro metabolic networks and highlights the utility of online monitoring approaches, especially using MS.

## Conclusions

Controlling the output of large ERNs requires models that map the reaction kinetics and the crosstalk within them effectively. Flow chemistry allows us to systematically explore the continuously changing reaction conditions. However, when the experimental setup is coupled to offline analysis methods, this exploration becomes less informative and thereby less efficient (see Figure S11). In this context, achieving real-time quantitative monitoring of the product mixture from large reaction networks in flow remained an outstanding analytical challenge.

We address this challenge by interfacing an ERN compartmentalized in a CSTR with an ion mobility–mass spectrometer. This interface enables us to gather real-time information about the metabolic flux of the ERN under changing conditions. Quantitative data are obtained by the generation of isotopically ^13^C-labeled metabolites in situ and the direct comparison of their ion intensities with nonisotopically labeled standards. The concentrations of two cofactors, ATP and NADH, could be respectively determined from isotopically labeled standards and online absorbance measurements. Using this interface, we quantified up to 8 metabolites simultaneously, in real time, from a reaction network built with 12 enzymes immobilized on hydrogel beads. As discussed above, in-flight fragmentation presents a possible drawback to MS approaches, although independent measurements of fragmentation yields can estimate metabolite concentrations, as demonstrated in this work for F6P and Lac. Notwithstanding this limitation, the data gathered using the MS interface remained remarkably informative and sufficient to train a model that mapped the overall dynamics of the entire network within a single OED iteration, simplifying the procedure to gain control over reactions within a CSTR.

The number of OED iterations required to train a model that can reliably control the reactions within an ERN is a function of the size of the network, the nonlinearity of the interactions within the network, and the resolution of the time course measurements. By addressing the latter, the active learning approach can be significantly improved, enabling us to predict reactor conditions with a single training data set in this case. This work presents the opportunity to expand the analytical toolbox, increasing both the throughput and observability for the analysis of enzymatic networks compared to offline methods. We envision that direct feedback could be established between the ERN in flow and the analytical instrument, enabling efficient testing of different topologies and allosteric terms in real time. Ultimately, this is required to design and control larger networks, where a combinatorial explosion of these potential “hidden” interactions can make them difficult to disentangle from one another, while their downstream effects make the network difficult to control. Novel analytical approaches that provide more informative data sets are essential for tackling this challenge.

## Experimental Section

### Production of Enzyme Beads

All free enzymes were purchased from Sigma-Aldrich, except for PGI, which was expressed and purified in-house from *Escherichia coli* (Table S1). Hydrogel beads were obtained following a procedure described previously.^18^ Immobilization is performed by rewetting the beads in Milli-Q, followed by activation of the carboxylic acid moieties by EDC/NHS coupling. The free enzyme in TRIS buffer (200 mM, pH 7.8) is then added (Table S2) for a 2 h coupling step. Sequentially, the beads were washed and centrifugated. This immobilization yielded active beads for all enzymes, except for GAPDH. The immobilization procedure for GAPDH is detailed in Supporting Information, along with additional information on the production of enzyme beads and activity assays.

### Flow Experiments

A custom-made CSTR (volume = 100 μL), made of poly(methyl methacrylate), was charged with the required volume of each enzyme bead (Tables S5–S9). The inlet and outlet of the reactor were sealed with Whatman Nuclepore polycarbonate membranes (10 μm pore size, cat. no. 10418406) to prevent the outflow of enzyme beads. We used Labm8 syringe pumps and HSW Plastipak 3-part syringes to dose inflows to the CSTR.

The outlet of the reactor was connected to a check valve (IDEX, CV-3301). Absorbance of the CSTR outflow was then continuously measured with an in-house 3D-printed flow cell, provided to us by Labm8, connected to an AvaLight 355 nm LED lamp. Absorbance between 340 and 360 nm was detected using an AvaSpec-2048 with 100 ms integration time and averaging for 8 scans. A Harvard PhD Ultra syringe pump was used to dispense the dilution line, which contained all analytical standards, with a flow rate of 87.5 μL·min^–1^. The total flow was then diverted between the mass spectrometer and a Restek RT-25020 back pressure regulator (BPR) connected to a waste line. The BPR provided a constant back pressure of two bar in the system. Detailed information about the setup can be found in Supporting Information. An overview of the experiments, including flow rates, bead compositions, and syringe solutions, can be found in Tables S5–S9.

### Mass Spectrometry

Ion mobility–MS (IMS–MS) experiments were performed with a timsToF instrument (Bruker, Germany) equipped with an ESI source. Ions were electrosprayed in negative mode with a source voltage of −3.5 kV, a nebulizer of 2.0 bar, a drying gas flow of 8 L·min^–1^, and a source temperature of 250 °C. Typical ion transfer voltages were quadrupole ion energy = −5 eV and collision energy = −8 eV. The mass range scanned by the ToF analyzer was *m*/*z* 50–1050. TIMS experiments were performed in N_2_ using the imeX Custom mode by scanning inverse ion mobilities from 0.35 to 1.3 V·s·cm^–2^, with a ramp time set at 550 ms. The accumulation time was set to 100 ms. The Bruker ESI needle was replaced with 15 cm long fused silica capillary tubing (Postnova Z-FSS-100190).

Ion chromatograms were extracted with a width of ±0.005 Da, and ion chromatograms for F6P/^13^C_6_-F6P and G6P/^13^C_6_-G6P were extracted for the mobility ranges 0.655–0.665 and 0.675–0.685 V·s/cm^2^, respectively (Figure S1). Raw ion intensities were normalized by the total ion current. To accurately determine the ion abundance of ^15^N-glutamic acid and NADH, the contributions of ^13^C isotopes of glutamic acid and NAD were removed according to Table S4. The data were then binned in 45 s intervals. Details about compound quantification and correction for in-flight fragmentation can be found in Supporting Information.

### Software and Modeling

The software itself is written in Python 3.8 (Python Software Foundation, Delaware, US). Code can be found at Huckgroup GitHub at http://github.com/huckgroup/OED archived with DOI: 10.5281/zenodo.10411170 (2023). The OED and fitting algorithm utilizes the AMICI solver, which is an ODE compilation package for C++ software which integrates with multiple tools.^43−47^ For more information on the theory behind OED, refer to eqs S1–S3. For more information on the model, refer to eqs S4–S8.

## Data Availability

Code can be found at Huckgroup GitHub at http://github.com/huckgroup/OED archived with DOI: 10.5281/zenodo.10411170 (2023). Raw ion intensities and notebooks used to generate the figures can be found at Huckgroup GitHub at https://github.com/huckgroup/IMS-MS_Glycolysis.

## Supporting Information Available

Full description of all materials and methods used, details of the OED algorithm, and details of software used (PDF)
