# Supplementary material for: Quantitative Online Monitoring of an Immobilized Enzymatic Network by Ion Mobility–Mass Spectrometry
Source: J Am Chem Soc. 2024 Jul 16;146(30):20778–87. doi: 10.1021/jacs.4c04218 (PMC11295183; doi:10.1021/jacs.4c04218)
Supplement: Supplementary file 1 — ja4c04218_si_001.pdf [file ja4c04218_si_001.pdf]

Quantitative online monitoring of an immobilized enzymatic network by ion mobility-mass spectrometry

Quentin Duez≠, Jeroen van de Wiel≠, Bob van Sluijs, Souvik Ghosh, Mathieu G. Baltussen, Max T. G. M. Derks, Jana Roithová, Wilhelm T.S. Huck*

Radboud University, Institute for Molecules and Materials, Heyendaalseweg 135, 6525 AJ, Nijmegen, The Netherlands.

SUPPORTING INFORMATION

Supplementary Tables – p2

Supplementary Figures – p5

Optimal experimental design and modelling glycolysis in flow – p9

Model of the glycolysis pathway – p9

Parameter estimation, model training and testing – p11

OED algorithm settings – p12

Simulation of undetected species – p12

Brief exploration of alternative optimization approaches – p13

Materials and methods – p14

- **Section 1 : Bead production – p14**
- **Section 2 : General immobilization protocol – p14**
- **Section 3 : GAPDH – SPAAC protocol for GAPDH immobilization – p14**
- **Section 4 : Qualitative enzyme activity assays – p14**
- **Section 5 : Preparation of stock solutions for analytical standards – p18**
- **Section 6 : Mass spectrometry – p18**
- **Section 7 : Setup of the experimental system – p19**
- **Section 8 : Compound quantification using isotopologues – p20**
- **Section 9 : Correction for in-flight fragmentation – p22**
- **Section 10 : NADH quantification – p24**

Overview of the experiments – p26

Software and data availability – p33

References – p33

SUPPLEMENTARY TABLES

Ion	Ion formula	Calculated mass (amu)	Measured mass (amu)	Δ (ppm)
**Glucose (*)**	$[C_6H_{11}O_6]^-$	179.0550	179.0547	1.68
**$^{13}C_6$-Glucose**	$[^{13}C_6H_{11}O_6]^-$	185.0751	185.0750	0.54
**Glucose/Fructose 6-phosphate (*)**	$[C_6H_{12}O_9P]^-$	259.0213	259.0206	2.70
**$^{13}C_6$-Glucose/Fructose 6-phosphate**	$[^{13}C_6H_{12}O_9P]^-$	265.0415	265.0410	1.89
**$^{13}C_6$-6-Phosphogluconolactone**	$[^{13}C_6H_{10}O_9P]^-$	263.0258	263.0251	2.66
**Fructose 1,6-biphosphate (*)**	$[C_6H_{13}O_{12}P_2]^-$	338.9877	338.9865	3.54
**$^{13}C_6$-Fructose 1,6-biphosphate**	$[^{13}C_6H_{13}O_{12}P_2]^-$	345.0078	345.0063	4.35
**$^{13}C_3$-Dihydroxyacetone phosphate / $^{13}C_3$-Glyceraldehyde 3-phosphate**	$[^{13}C_3H_6O_6P]^-$	171.9997	171.9997	0.00
**$^{13}C_3$-1,3-Bisphosphoglycerate**	$[^{13}C_3H_7O_{10}P_2]^-$	267.9610	/	/
**3/2-Phosphoglycerate (*)**	$[C_3H_6O_7P]^-$	184.9846	184.9842	2.16
**$^{13}C_3$-3/2-Phosphoglycerate**	$[^{13}C_3H_6O_7P]^-$	187.9946	/	/
**Phosphoenolpyruvate (*)**	$[C_3H_4O_6P]^-$	166.9740	166.9740	0.00
**$^{13}C_3$-Phosphoenolpyruvate**	$[^{13}C_3H_4O_6P]^-$	169.9841	/	/
**^{13}C-Pyruvate (*)**	$[^{13}C_1C_2H_3O_3]^-$	88.0110	88.0115	-5.68
**$^{13}C_3$-Pyruvate**	$[^{13}C_3H_3O_3]^-$	90.0177	90.0182	-5.55
**Lactate (*)**	$[C_3H_5O_3]^-$	89.0233	89.0236	-3.37
**$^{13}C_3$-Lactate**	$[^{13}C_3H_5O_3]^-$	92.0334	92.0338	-4.35
**ATP**	$[C_{10}H_{13}N_5O_{13}P_3Na]^{2-}$	263.4805	263.4737	25.81
**$^{13}C_{10}$-$^{15}N_5$-ATP (*)**	$[^{13}C_{10}H_{13}{}^{15}N_5O_{13}P_3Na]^{2-}$	270.9898	270.9830	25.09
**ADP**	$[C_{10}H_{14}N_5O_{10}P_2]^-$	426.0210	426.0190	4.69
**NAD**	$[C_{21}H_{26}N_7O_{14}P_2]^-$	662.1007	662.0967	6.04
**NADH**	$[C_{21}H_{28}N_7O_{14}P_2]^-$	664.1164	664.1119	6.78
**Glutamic acid (*)**	$[C_5H_8NO_4]^-$	146.0453	146.0448	3.42
**^{15}N-Glutamic acid**	$[C_5H_8{}^{15}NO_4]^-$	147.0413	147.0420	-4.76

Table S1. Calculated and measured *m/z* for the metabolites detected in the experiments described in the main text. The compounds marked with an asterisk (*) correspond to standards infused in the dilution line. The mass accuracy for ATP is relatively poor compared to other compounds, nevertheless the detection of both unlabelled and $^{13}C_{10}$-$^{15}N_{15}$-labelled ATP with a similar mass shift supports our assignment. The compounds marked with a '/' have not been detected.

Enzyme	Abbreviation	Product no.	Loading (U/mg)
**Hexokinase**	HK	H4502	200
**Glucose phosphate isomerase**	GPI	P5381	100
**Glucose-6-phosphate dehydrogenase**	G6PDH	G8529	100
**Phosphofructokinase**	PFK	F0137	50
**Aldolase**	Ald	A8811	50
**Triose phosphate isomerase**	TPI	T6258	100
**Glyceraldehyde-3-phosphate dehydrogenase**	GAPDH	G2267	40[a]
**3-Phosphoglycerate phosphokinase**	PGK	P7634	100
**Phosphoglycerate isomerase**	PGI	- [b]	20
**Enolase**	Eno	E6126	100
**Pyruvate kinase Subtype M2**	PKM2	SAE0021	10
**Pyruvate kinase Subtype III**	PK III	P9136	- [c]
**Lactate dehydrogenase**	LDH	L1254	80

Table S2. Overview of all different enzyme beads used in this work, most free enzymes were purchased from Sigma-Aldrich and immobilized using the general immobilization procedure. a) GAPDH was immobilized using the SPAAC click reaction. b) PGI was expressed and purified in-house from E. coli. c) PK III was only used in free form to evaluate the activity of other immobilized enzymes (See **Section 4**)

Analyte	Nucleus	$[A]_T$ (mM)	$[A]_C$ (mM)
**ADP**	^{31}P	100	95.5
**ATP**	^{31}P	100	91.4
**NAD$^+$**	^{31}P	100	92.0
**G6P**	^{31}P	100	89.6
**F6P**	^{31}P	100	77.1
**FBP**	^{31}P	100	55.9
**3PG**	^{31}P	100	110.9
**PEP**	^{31}P	125	93.3
**Glucose**	^{1}H	100	79.6
**Fructose**	^{1}H	100	92.9
**Pyruvate**	^{1}H	100	98.4
**$^{13}C_1$ -Pyruvate**	^{1}H	100	91.2
**Lactate**	^{1}H	125	120.4
**Potassium Glutamate**	^{1}H	1000	1103.4
**$^{15}N_1$-Glutamic acid**	^{1}H	25	26.4

Table S3. Recalculated concentrations of stock solutions for each of the analytical standards based on ^{1}H or ^{31}P NMR. Hexamethylphosphoramide (HPMA) and trimethylsilylpropanoic acid (TMSP) were used as standards for ^{31}P and ^{1}H NMR, respectively. In case of FBP, glucose and fructose different isomers were detected which we presumed to be the respective α or β anomer, or the open form. In such cases, the integrals of the same nucleus are summed. $[A]_T$ stands for theoretical stock concentration and $[A]_C$ for corrected stock concentration (see **Equation S9**).

SUPPLEMENTARY FIGURES

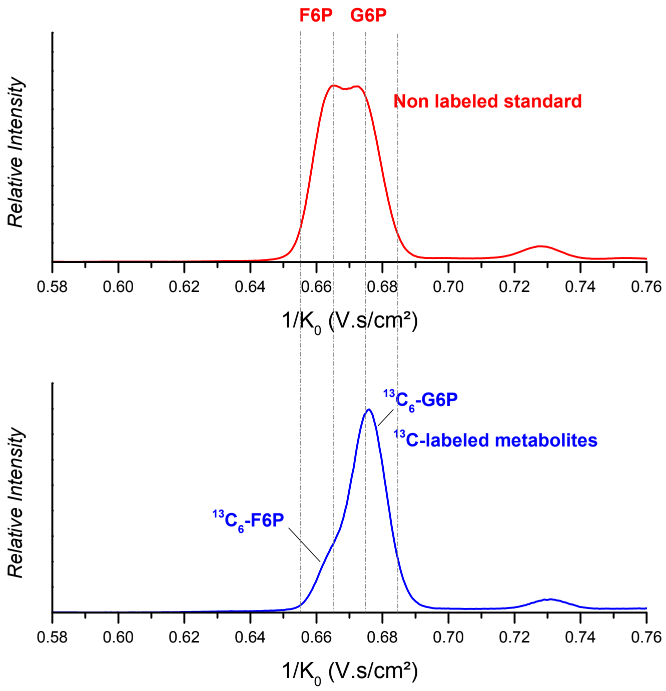

Figure S1. Ion mobility separation of glucose 6-phosphate and fructose 6-phosphate isomers. Top: Non labelled standards added to the dilution line, and Bottom: ^{13}C-labelled metabolites produced in the CSTR. The ranges highlighted between vertical bars correspond to the inverse mobility ranges used to extract the intensities of F6P and G6P. The ions detected between $1/K_0$ 0.72-0.74 V.s/cm² correspond to isobaric ions.

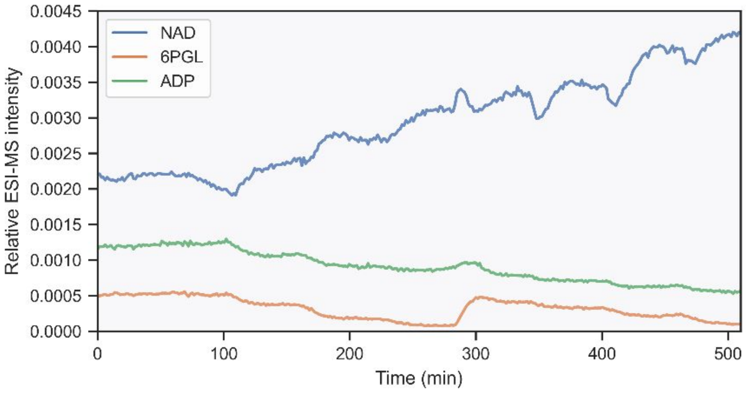

Figure S2. Relative ESI-MS intensities of NAD, 6PGL and ADP in the experiment shown in **Figure 2** of the main text.

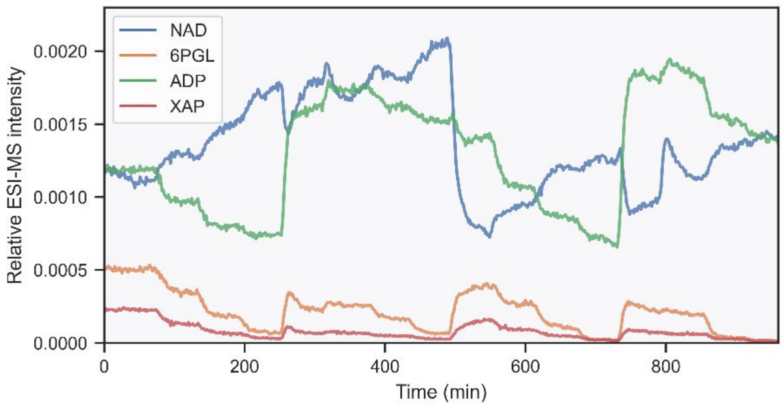

Figure S3. Relative ESI-MS intensities of NAD, 6PGL, ADP and XAP (GAP and DHAP) in the experiment shown in **Figure 3a** of the main text, starting from $^{13}C_6$-glucose.

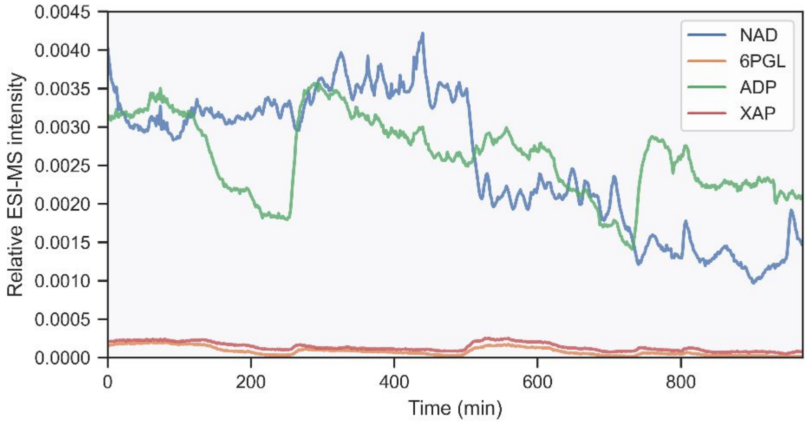

Figure S4. Relative ESI-MS intensities of NAD, 6PGL, ADP and XAP (GAP and DHAP) in the experiment shown in **Figure 3b** of the main text, starting from $^{13}C_6$-fructose.

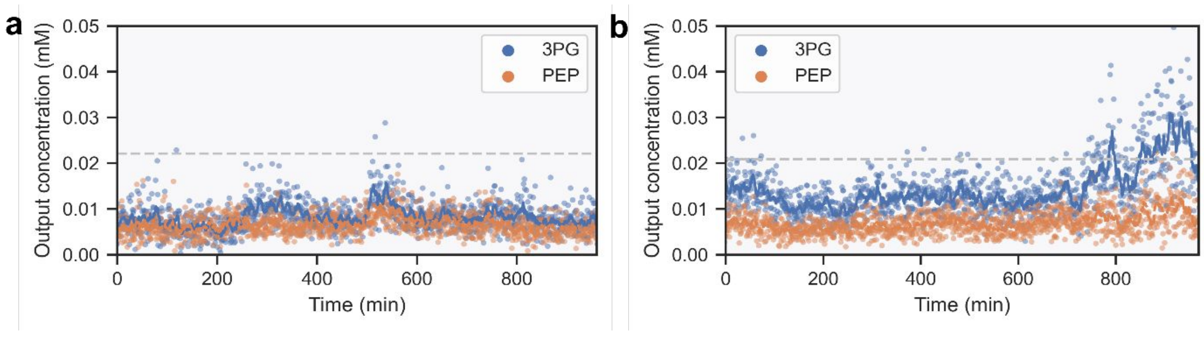

Figure S5. Estimation of the concentration associated with the intensity of the background noise detected at the *m/z* of 2/3PG and PEP for the experiments shown in **Figure 3**, starting from **a.** $^{13}C_6$-glucose and **b.** $^{13}C_6$-fructose. Dots correspond to binned data and lines correspond to rolling averages (n = 10). We estimated the lower bounds of detection as follows: $L = \overline{C_0} + 1.645.S(C_0)$, with C_0 being the concentration associated with the noise, and $S(C_0)$ being the standard deviation of the concentration associated with the noise. The respective values for **a.** and **b.** are 22 and 21 µM respectively, as highlighted by a horizontal dashed line. We thus estimate the lower bounds of detection at ~20 µM.

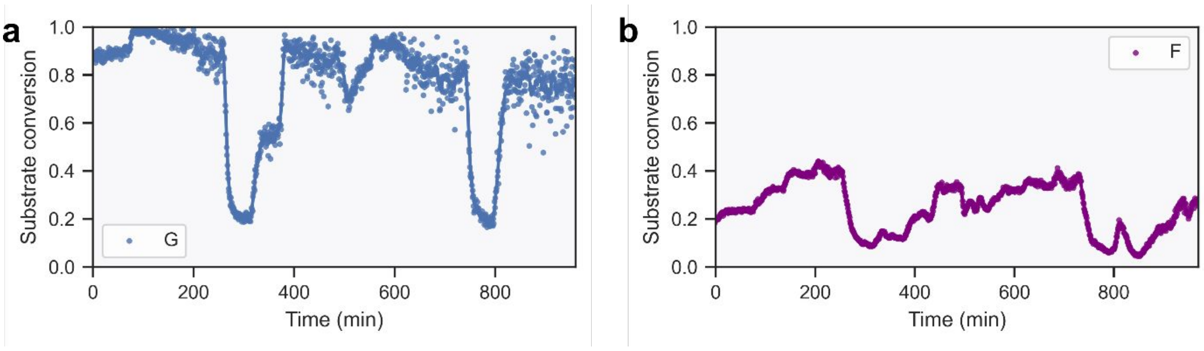

Figure S6. Observable substrate conversion for the experiments shown in **Figure 3**, starting from **a.** $^{13}C_6$-glucose and **b.** $^{13}C_6$-fructose. Dots correspond to binned data and lines correspond to rolling averages (n = 10). The substrate conversion is defined as the ratio between the measured concentration of observed product metabolites and the total concentration of substrate and observed product metabolites (G/F, G6P, F6P, FBP, Lac, Pyr).

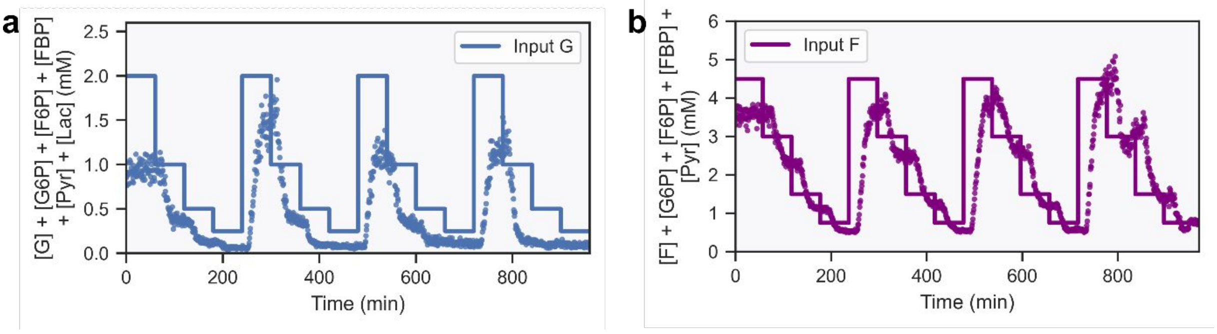

Figure S7. Comparison between the summed concentrations of observable substrates and product metabolites (G/F, G6P, F6P, FBP, Pyr and Lac) for the experiments shown in **Figure 3** and the input concentration of **a.** $^{13}C_6$-glucose and **b.** $^{13}C_6$-fructose.

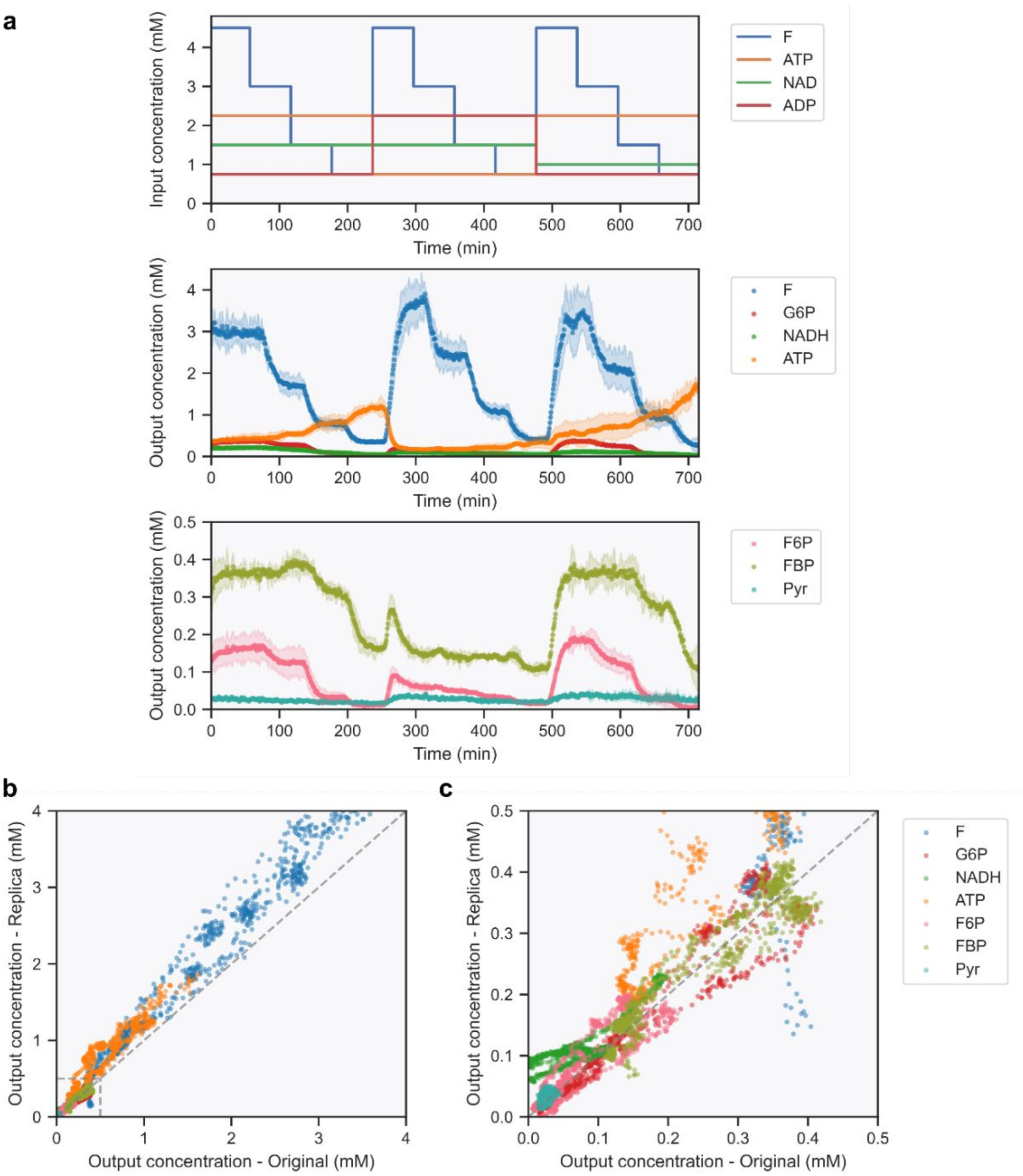

Figure S8. a. Output concentrations averaged across two datasets for the same input modulations, shown in **Figure 3b** starting from $^{13}C_6$-fructose. The standard deviation is represented by light areas around the averaged concentrations. **b, c.** Comparison of the output concentrations measured for the original experiment and the replica. The grey line represents y=x, a perfect correlation. The datasets have been measured on two different days with different CSTRs, fresh bead mixtures and stock solutions.

OPTIMAL EXPERIMENTAL DESIGN AND MODELLING THE GLYCOLYSIS IN FLOW

The model of the glycolysis pathway is defined by a series of ordinary differential equations defined in **Equation S1**.

$$\frac{dX}{dt} = f(x,t,u,p) \tag{S1}$$

Where ***X*** represent the states in the set of ODEs ***x*** the individual state, ***t*** the time, ***u*** the control parameter and ***p*** the kinetic parameters. Most optimal experimental design problems utilize the Fisher information matrix.[1-3] This matrix can be computed from the sensitivities of the parameters p towards the observed species x according to **Equation S2**.

$$\frac{d}{dt}\frac{\partial x}{\partial p_i} = \frac{\partial f(x,u,p)}{\partial x}\frac{\partial x}{\partial p_i} + \frac{\partial f(x,u,p)}{\partial p_i} \quad for\ i = 1,\dots,N_p \tag{S2}$$

Where $\frac{\partial f(x,u,p)}{\partial x}$ is the Jacobian, $\frac{\partial x}{\partial p_i}$ the sensitivity coefficients and $\frac{\partial f(x,u,p)}{\partial p_i}$ the parameter sensitivities. Note, we end up with an additional $\boldsymbol{N_p} * \boldsymbol{N_x}$ number of sensitivity ODEs.[6] By differentiating the original ODEs with respect to the parameters we effectively map how much each parameter contributes to the rate of change in the observed species. The sensitivity matrix can subsequently can be obtained by stacking these sensitivities towards different observables at different times according to:

$$\boldsymbol{S} = \begin{bmatrix} \frac{\partial y_{j1}}{\partial p_1}(t_1) & \cdots & \frac{\partial y_{j1}}{\partial p_{N_p}}(t_1) \\ \vdots & & \vdots \\ \frac{\partial y_{j1}}{\partial p_1}(t_{N_t}) & \cdots & \frac{\partial y_{j1}}{\partial p_{N_p}}(t_{N_t}) \\ \frac{\partial y_{j2}}{\partial p_1}(t_1) & \cdots & \frac{\partial y_{j2}}{\partial p_{N_p}}(t_1) \\ \vdots & & \vdots \\ \frac{\partial y_{j2}}{\partial p_1}(t_{N_t}) & \cdots & \frac{\partial y_{j2}}{\partial p_{N_p}}(t_{N_t}) \end{bmatrix}$$

Where $\boldsymbol{y}$ is are the subset of observable states ***x***, $\boldsymbol{p}$ the parameter and $\boldsymbol{t}$ the time point of the observation up to $\boldsymbol{t_{N_t}}$ and ***j*** the index corresponding to individual experiments. Thus, each column is a parameter, each row is a time point, where new observables and experiments are added to columns.

The Fisher information matrix is subsequently computed according to **Equation S3**.[2-4]

$$\boldsymbol{F} = \boldsymbol{S}\boldsymbol{S}^T \tag{S3}$$

According to Cramer-Rao, the inverse of ***F*** provides a lower bound to the parameter covariances.[5] This ***FIM*** is local, thus the computed information criterion needs a model that has some prior knowledge of the system. Practically we are using the sensitivities of the parameters we are trying to estimate as an input for ***F***, thus if the initial parameter values used in the design of the pulse sequence are very far from their true value so will the accuracy of the computed information criterion (resulting in less informative data).[6] Therefore we fit the model to the data of the small subnetwork presented in **Figure 2** in the main text. For this work we utilized D-optimal design, calculating the determinant of ***F***, where $\boldsymbol{D} = \mathbf{max}_{\boldsymbol{D}}\ (\boldsymbol{F})$ (E-optimality is also an option, computing the smallest eigenvalue of ***F*** which represents the direction with the least variance between sensitivity).[2, 7] For more information on OED in this explicit context see ref 9 and ref 10.[8, 9]

MODEL OF THE GLYCOLYSIS PATHWAY

In previous work we have shown that the reactions for enzymes coupled to beads can be modelled by a generalized Hill equation where ***V*** for all the bi-substrate reactions is given by[10]

$$V = \frac{[S1][S2][Enzyme\ ]k_{cat}}{Km_1 Km_2\left(1 + \frac{[S1]}{Km_1}\right)\left(1 + \frac{[S2]}{Km_2}\right)} \tag{S4}$$

Where [***S1***] and [***S2***] are the substrate concentrations, [enzyme] the concentration of beads multiplied by their activity, $\boldsymbol{k_{cat}}$ the catalysis rate of the enzymes. the flux for a reversible reaction subsequently within a microfluidic flow reactor becomes

$$\frac{dX_i}{dt} = V_{Forward} - V_{reverse} + Kf_{in,I}X_{in,stock} - Kf * X_i \quad \text{(S5)}$$

Where the forward and reverse reactions are noted by V (with different kinetic rates), and the inflow $\boldsymbol{Kf_{in,I}}$ and outflow $\boldsymbol{Kf}$ are proportional to the stock concentration and the concentration in the reactor respectively. The inflow rate of input substrates is modelled and accounts for individual syringes connected to the CSTR reactor. Thus, the concentration of the input substrate in a syringe is larger than the final concentration in the reactor (as multiple syringes are connected to the CSTR), and is modelled according to:

$$[X_{ss}] = X_{in,stock}\frac{Kf_{in,I}}{kf} \quad \text{(S6)}$$

Where $[\mathbf{X_{ss}}]$ is the concentration of a single substrate that flows into the reactor at steady state. $\boldsymbol{Kf_{in,I}}$ is the flowrate of a specific substrate/syringe combination that flows into the reactor. $\boldsymbol{X_{in,stock}}$ is the stock concentration of the substrate. Each $\boldsymbol{Kf_{in,I}}$ i.e., substrate/syringe combination contributes to the total flowrate $\boldsymbol{Kf}$. The total flow rate (flow out of the reactor) that applies to all species is defined as: the sum of all $\boldsymbol{Kf_{in,I}}$.

$$kf = \frac{\sum_{i=1}^{i} Kf_{in,I}}{\text{volume}} \quad \text{(S7)}$$

Note that the generalized Hill equation is very similar to the reaction kinetics described in Cook and Cleland's Enzyme kinetics,[11] where the V for rapid equilibrium random reaction is given by

$$V = \frac{[S1][S2][Enzyme\,]k_{cat}}{K_{ai}K_b + K_b[S1] + Ka[S2] + [S1][S2]} \quad \text{(S8)}$$

In our system we assume $\boldsymbol{K_{ai}} = \boldsymbol{K_a}$ an assumption that holds for a large ensemble of partially observed systems. This is described in Rohwer et al.[10] and Cook and Cleland page 83[11] and in our previous work where we tested different rate law variants for enzymes coupled to beads.[9] The allosteric inhibition reaction (see main text, **Figure 1**) is obtained by multiplying the denominator of V in **Equation S4** by $A = (\frac{[i]}{ki} + 1)$. The SBML file of the model is uploaded to the huckgroup Github, http://github.com/huckgroup/OED.

PARAMETER ESTIMATION, MODEL TRAINING AND TESTING

In previous work we showed that we can balance parameter identifiability with model complexity and its predictive power.[9] We do not need to know individual rates, these rates need to map onto the conversion fluxes sufficiently well to predict new conditions in the reactor and the remaining uncertainty needs to be manageable. To that end, we opted to use the experiment shown in **Figure 3a** as test data, here, the stock solutions in the syringe and the enzyme concentrations differ from the optimally designed training data, representing a new set of conditions (other than variations in time dependent inputs).

We tested if the model could be trained on the test dataset, to assess if these data could be mapped by the model. **Figure S9** shows that the model cannot match Lac measurements, indicating a possible measurement error cannot be ruled out, especially given how many degrees of freedom this model has (56 parameters). As discussed in the main text, the assumption of consistent fragmentation ratios for the fragmentation of G to Lac, or an isomer of Lac, is probably not accurate. For G6P and F6P the model and data did converge better compared to the prediction in the main text. This likely means that the OED process did not map the low ATP concentration regimes adequately, whereby inaccurate parameter sensitivities within this regime cause the predictions to diverge. Regardless, as a proof of principle the predictions are remarkably close, notably, when compared to prior work where the error were significantly larger (factor >10).[9] Finally to quantify the goodness of fit between the model and test data or model and training data we log2 normalized the concentrations prior to calculating the root mean squared error.

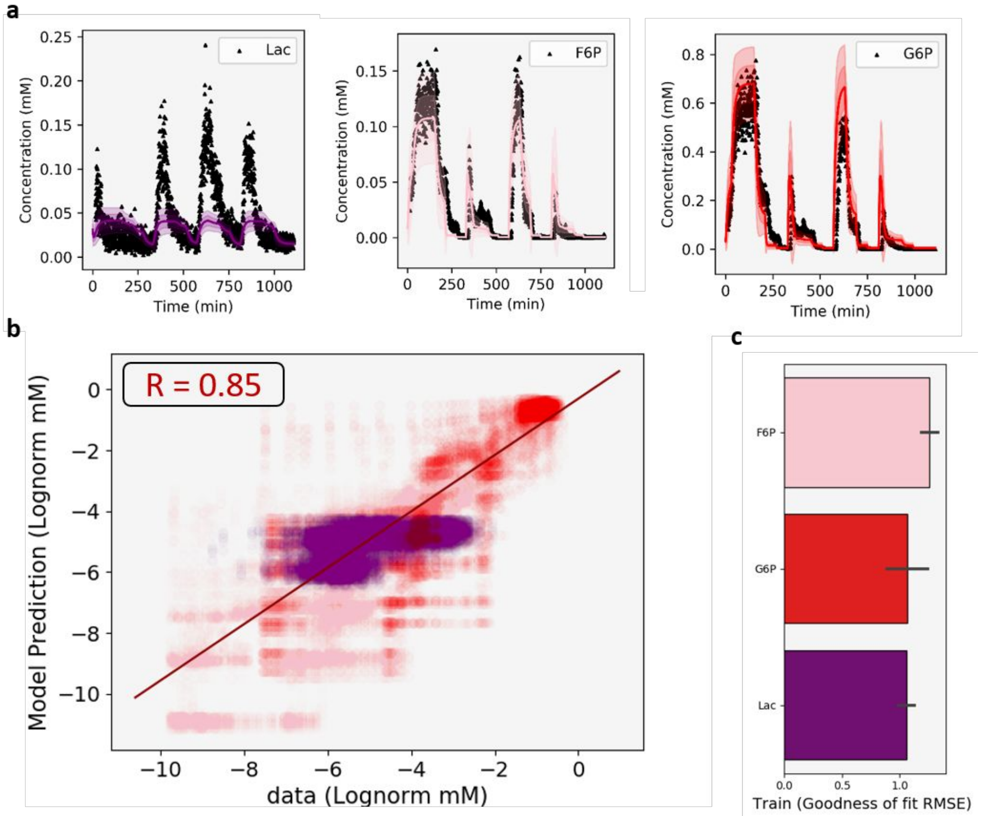

Figure S9. *a) The fit of the model to the test data indicates that G6P and F6P concentrations can be approximated, indicating it is not a fundamental limitation of the model. Thick line represents the mean, shaded areas 1 and 2 standard deviations between simulations respectively. b) shows the scatter plot for R score computation, LAC remains an outlier with respect to the R2 plot and with prediction with standard deviation. c) for LAC, RMSE scores are similar to F6B and G6P. Yet, the concentration difference between the lowest and highest reported value differed less for LAC, thus so the log normalized RMSE value is less similar.*

Both here and in the main text, the concentrations below 10 µM are not taken into account for log2 transformed data, because these concentrations cannot be accurately determined experimentally (see **Figure S5**). Regardless, to ensure we give a comprehensive overview of testing (and training) we opted to show the raw data with the prediction including 2 standard deviations in a time course plot, an R-score between predicted and measured concentrations and a bar plot with the RMSE scores for comparison. Note that it is hard to compare the parameter estimates of enzymes on beads to those of free enzymes reported in literature, because we do not know the exact

concentration of active enzymes after coupling them to beads. Thus, this report primarily focusses on training a model that can predict ERN outcomes. The effective parameter estimates are added in a Source Data file.

OED ALGORITHM SETTINGS

The OED algorithm is characterized by agents that move across a fitness landscape. The agents consist of the model(s) and the datasets an OED optimization problem. If the user supplies datasets, then the algorithm will fit the model to these datasets by allowing the kinetic parameters to mutate for each of the agents (moving them across the landscape). Every 15 iterations, unfit agents are re-initialized next to the fittest agents. The same applies for an optimal experimental design problem, however, instead of fitting the model to a datasets, the agents mutate the control parameters for selected time windows, the aggregate fitness score is subsequently defined by computing the Fisher-D criterion of the experiment (**Equation S3**). For more information we refer the reader to ref 9 and ref 10.

To fit the data we needed 30 agents per iterations to ensure convergence and 500 iterations. The algorithm utilizes a multi-start approach using Latin hypercube sampling to sample 1500 parameter sets. The 2 datasets (small network and OED experiment) are fit simultaneously. For the optimal experimental design 100 random pulse experiments were generated and optimized further, 10 were optimized a single experiment was chosen. The boundaries of the parameter values span 6 orders of magnitude (0.001,50). The OED experiments were constrained by 3 flowrate stages, consistent flow patterns reduce noise and the odds of reactor failure. For more information on this approach we refer the reader to ref 10, where the consequences of these decisions are explored in more detail.

SIMULATION OF UNDETECTED SPECIES

As discussed in the main text, some species (PEP, BPG, 2/3PG) were not detected in the experiments shown in **Figure 3**. We suspected that these species were present with low concentrations in solution. To confirm we used the trained model to simulate their concentrations in the experiment from **Figure 3a**. Gratifyingly, PEP, BPG, DHAP, PG2 and PG3 were all predicted to be at very low concentration regimes for the duration of the experiments (**Figure S10** - comparison to experiment shown in **Figure 3a** of main text).

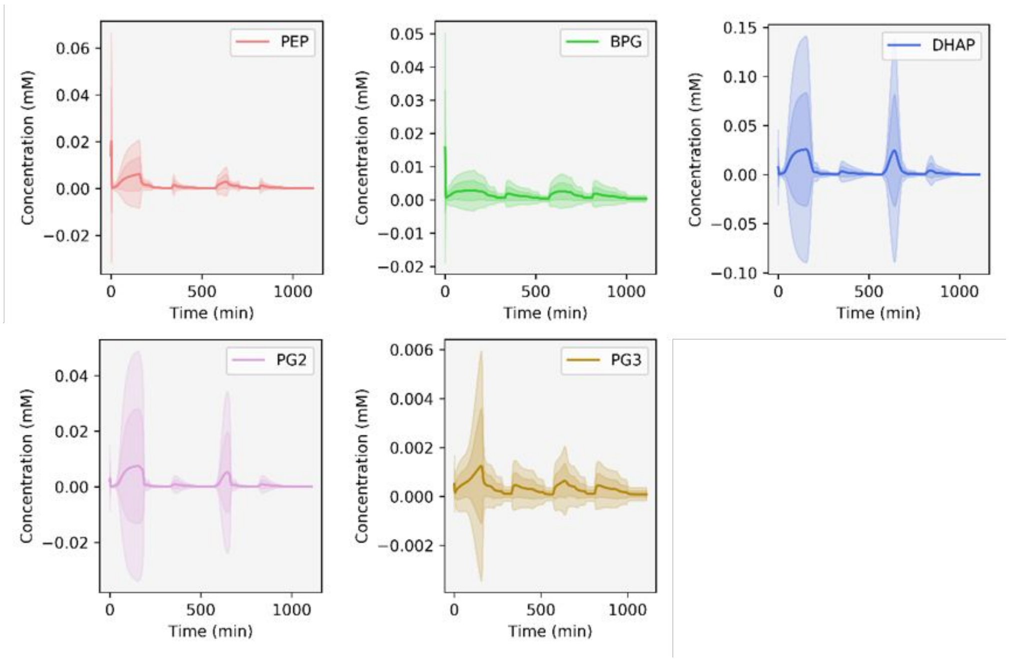

Figure S10. *Simulation (N = 10) and resulting predicted time course of species PEP, BPG, DHAP, PG2 and PG3. All predicted to be in a low concentration regime. Thick line represents the mean, shaded areas 1 and 2 standard deviations between simulations respectively (Seaborn plotting allows negative concentrations for STD, no negative concentrations present in simulation).*

BRIEF EXPLORATION OF ALTERNATIVE OPTIMIZATION APPROACHES

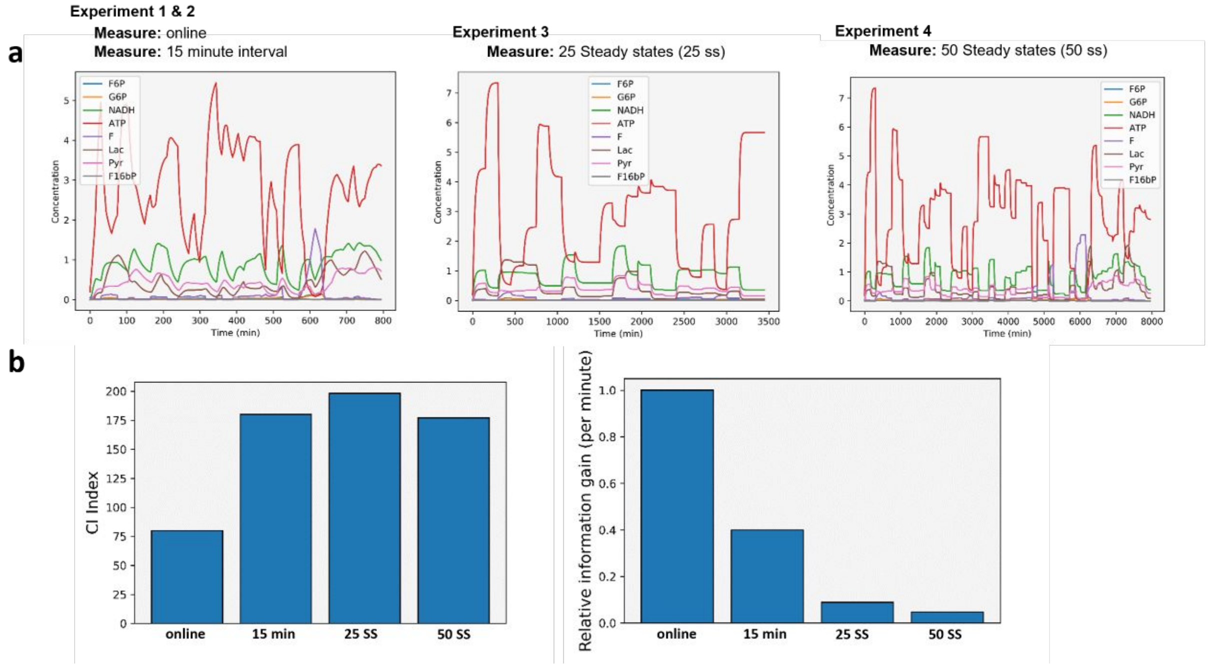

***Figure S11. a**. Simulation of the four in silico experiments for which the collinearity index (CI) was computed. Experiments 1 and 2 show dynamic experiments where the system never reaches a steady state. In experiment 1, measurements are taken every minute; in experiment 2, measurements are taken every 15 minutes. For experiment 3, we allow the system to reach a steady state and measure the steady states of the observables, with a total of 25 states measured. In experiment 4, 50 steady states are measured. **b.** Shows the computed collinearity index (CI) for each experiment: the left panel indicates that a lower CI score equates to more information about the parameters being available in the data. The right panel shows the amount of information gained for every minute the experimental setup runs, computed by taking the relative differences between the online CI score and multiplying them by the relative differences between the experimental times (note that this is a rough, indicative analysis).*

Using online dynamic data to train a model is more efficient than any other approach, specifically previously reported steady-state data points[12, 13] or offline measurements of dynamic data.[9] To explore this, we performed a rough analysis where we computed the CI index for four in silico datasets (**Figure S11a**; for more information on the CI index, we refer the reader to reference[14]). These datasets vary either by the type of experiment or the time interval with which measurements are taken, namely:

- Steady-state data of the glycolysis network containing 25 data points.
- Steady-state data of the glycolysis network containing 50 data points.
- Dynamic data containing data points measured at 15-minute intervals (chosen for comparison since it also numbers 50 data points).
- A dataset with data points consisting of frequent (online) measurements (per minute).

The CI index, or collinearity index, denotes the degree to which parameters are collinear. It corresponds to the square root of the inverse of the smallest eigenvalue of F after performing a single value decomposition (E-Fisher information criterion instead of D-Fisher). A lower score equates to less collinearity between parameters, meaning the parameters are more identifiable.

In the analysis, we note that measuring 50 steady states results in a better CI score than measuring 25 (**Figure S11b**, left panel). This score is similar to dynamic data where we only measure at 15-minute intervals (resulting in the same number of final data points). All perform significantly worse than online measurement dynamic data, especially when considering the duration of these experiments. When we take the CI scores as a proxy for ‘information’ and translate these into relative information gained per minute of experiment, the steady-state experiments perform considerably worse (**Figure S11b**, right panel).

MATERIALS AND METHODS

1. Bead production. Lyophilized "empty" hydrogel beads consisting of 9.6% (w/v) acrylamide, 0.4% (w/v) N,N'-methylenebisacrylamide and 0.5% (w/v) acrylic acid were obtained following a procedure described previously.[12]

2. General immobilization protocol. Immobilization is performed by re-wetting the beads in Milli-Q (30 µL/mg of lyophilized beads). Subsequent addition of EDC (100 mM) and NHS (100 mM) activates the surface and increases the volume to 150 µL/mg. The solution is left to react for 30 minutes on a roller bank, after which the beads are centrifuged at 5000 x g for 3 min. The supernatant is carefully pipetted off. The activated beads are washed 3 times by adding Milli-Q, vortexing, centrifuging and removing the supernatant.

To this suspension, the free enzyme in TRIS buffer (200 mM, pH 7.8) is added, once again bringing the total volume to 150 µL/mg (**Table S2**). All enzymes purchased as suspension in ammonium sulphate were first centrifuged at 5000 x g for 3 minutes to remove the ammonium sulphate before being dissolved in TRIS buffer. The tube was put on the roller bank for another 2 h coupling step. Sequentially, beads were washed 8 times by adding Milli-Q, centrifuging and removing the supernatant. This immobilization yielded active beads for all enzymes, except for GAPDH.

3. GAPDH – SPAAC protocol for GAPDH immobilization. Immobilization is performed by re-wetting the beads in Milli-Q (30 µL/mg of lyophilized beads). Subsequent addition of EDC (100 mM) and NHS (100 mM) activates the surface and increases the volume 150 µL/mg. The solution is left to react for 30 minutes on a roller bank, after which the beads are centrifuged at 5000 x g for 3 min. The supernatant is carefully pipetted off. The activated beads are washed 3 times by adding Milli-Q, vortexing, centrifuging and removing the supernatant. After this, the azido-amine in Milli-Q (100 mM) was added to the beads, and were left to react for an additional 30 minutes. After repeating the washing steps with MilliQ, azido beads are afforded.

The enzymes are then functionalized with dibenzocyclooctyne-N-hydroxysuccinimidyl ester (DBCO-NHS). 400 units (4.21 mg, 29.2 nmol, 1 equivalent) of GAPDH are weighed and dissolved in Milli-Q, yielding a 400 u/mL solution. We then prepare an aqueous solution 1 mM of DBCO-NHS with 30% (v/v) DMSO, of which 58.5 µL (58.5 nmol, 2 equivalents) is added to the enzyme solution. The reaction is left for 30 minutes, after which the DBCO-enzyme solution is added to the azido beads. The resulting suspension is then washed according to the general procedure to yield the GAPDH beads.

4. Qualitative enzyme activity assays. All reactions were conducted in 200 mM TRIS HCl (pH 7.8), 20 mM KCl, 10 mM $MgCl_2$, 5 mM $K_XH_XPO_4$ and 0.5 mM $MnCl_2$. Activity assays were conducted either using plate reader, or using nucleotide HPLC (for 3PGK beads). The activity of TPI beads was not measured.

Plate reader

A solution of 100 µL was prepared in a GreinerBioOne 96 well transparent plate. The absorbance of the reporter metabolite NADH was measured at λ = 340 nm for a period of up to 60 minutes. Since beads affect the light transmission, these measurements indicate activity, but are not suitable for quantitative activity determination.

HK

**HK**	**5 µL**
**G6PDH (free)**	1 u/µL
**Glucose**	1 mM
**ATP**	2 mM
**NAD⁺**	2 mM

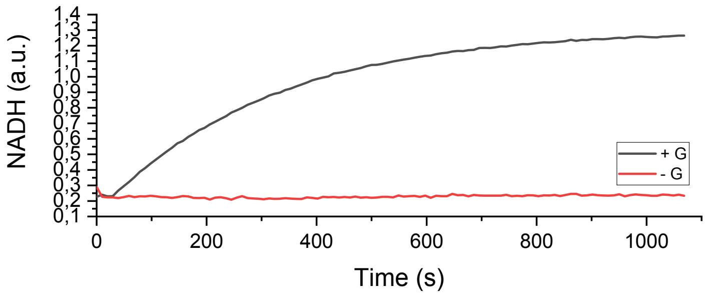

Figure S12. Activity assay of HK beads. Free G6PDH converts G6P to 6PGL, thereby leading to an increase in NADH concentration.

GPI

**GPI**	**5 µL**
**HK (free)**	1 u/µL
**G6PDH (free)**	1 u/µL
**Fructose**	1 mM
**ATP**	2 mM
**NAD⁺**	2 mM

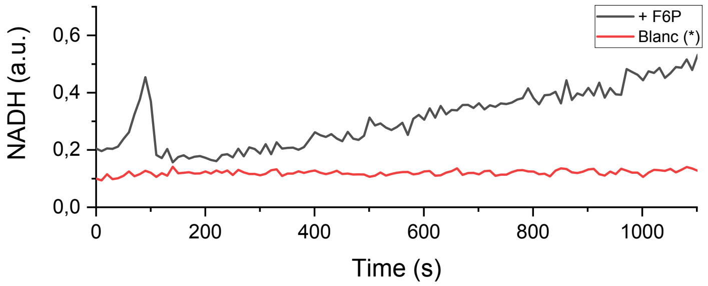

Figure S13. Activity assay of GPI beads. Free HK and G6PDH were added to observe an increase in NADH concentration, upon conversion of G6P to 6PGL. * The blanc line is from the Aldolase assay and meant to guide the eye.

PFK

**PFK beads**	**5 µL**
**PK III (free)**	1 u/µL
**LDH (free)**	1 u/µL
**F6P**	1 mM
**ATP**	2 mM
**PEP**	2 mM
**NADH**	2 mM

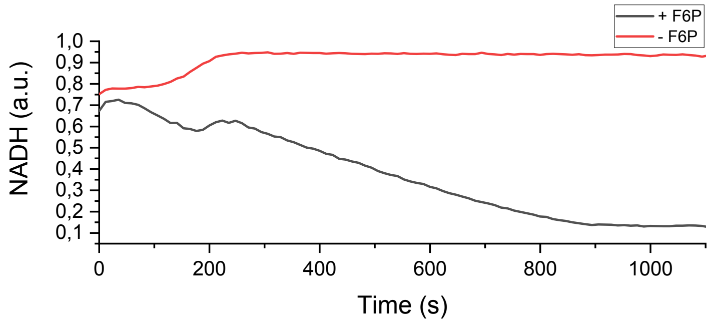

Figure S14. Activity assay of PFK beads. The PFK beads convert F6P in FBP, which produces ADP. The ADP is used by the free PK III to convert PEP in Pyr, followed by a conversion to Lac by free LDH, which depletes the concentration of NADH.

G6PDH

**G6PDH beads**	**5 µL**
**G6P**	1 mM
**NAD⁺**	2 mM

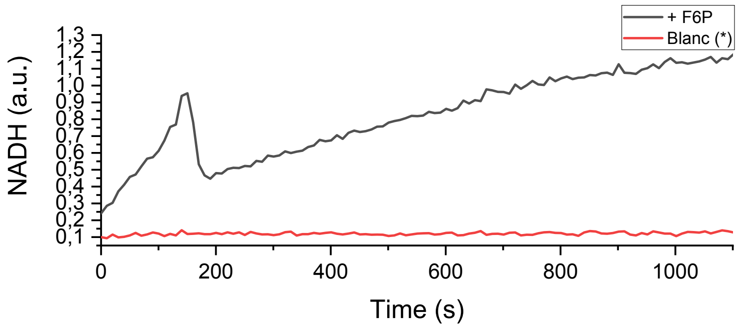

Figure S15. Activity assay of G6PDH beads. An increase in NADH concentration is observed upon conversion of G6P to 6PGL. * The blanc line is from the Aldolase assay and meant to guide the eye.

Aldolase

**Ald beads**	**5 µL**
**GAPDH (free)**	1 u/µL
**PGK (free)**	1 u/µL
**FBP**	1 mM
**ADP**	2 mM
**NAD⁺**	2 mM

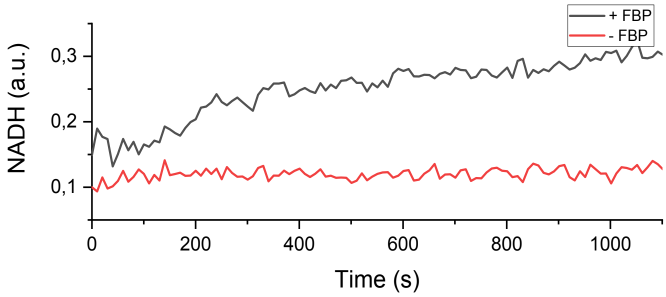

Figure S16. Activity assay of Aldolase beads. FBP is converted to GAP/DHAP by the Ald beads, which react then with free GAPDH, yielding an increase of NADH concentration. PGK is added in excess to drive the equilibrium of GAPDH towards BPG.

GAPDH

**GAPDH beads**	**5 µL**
**Ald (free)**	1 u/µL
**PGK (free)**	1 u/µL
**FBP**	1 mM
**ADP**	2 mM
**NAD⁺**	2 mM

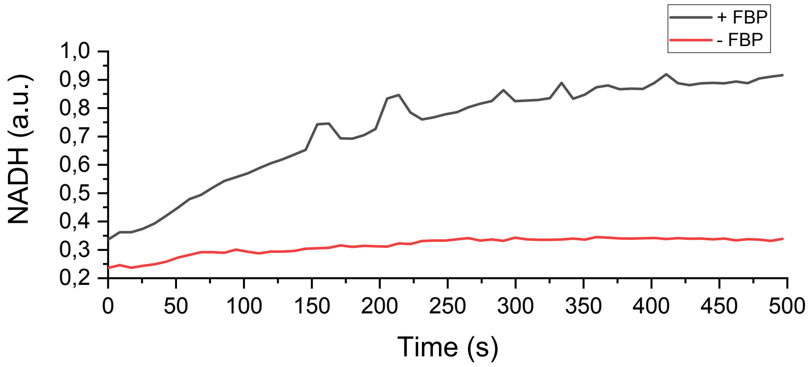

Figure S17. Activity assay of GAPDH beads. FBP is converted to GAP/DHAP by the free Ald, which react then with GAPDH beads, yielding an increase of NADH concentration. PGK is added in excess to drive the equilibrium of GAPDH towards BPG.

PGI

**PGI beads**	**5 µL**
**Eno (free)**	1 u/µL
**PK III (free)**	1 u/µL
**LDH (free)**	1 u/µL
**3PG**	1 mM
**ADP**	2 mM
**NADH**	2 mM

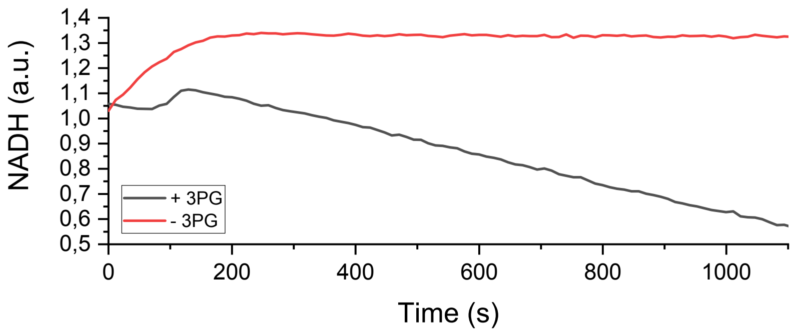

Figure S18. Activity assay of PGI beads. 3PG is converted to 2PG by the PGI beads, and then subsequently to PEP, Pyr and Lac by the free Eno, PK and LDH respectively. The NADH consumption, upon conversion from Pyr to Lac, is observed when 3PG is added.

Eno

**Eno beads**	**5 µL**
**PGI (free)**	1 u/µL
**PK III (free)**	1 u/µL
**LDH (free)**	1 u/µL
**3PG**	1 mM
**ADP**	2 mM
**NADH**	2 mM

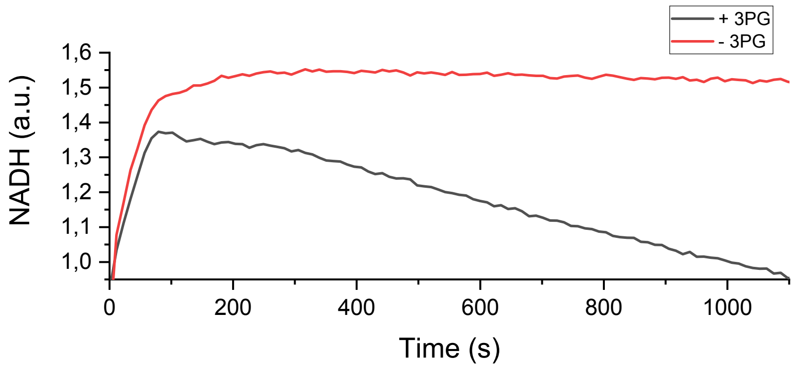

Figure S19. Activity assay of Eno beads. 3PG is converted to 2PG by free PGI, and then subsequently to PEP by Eno beads. PEP is converted to Pyr and Lac by the free PK and LDH respectively. The NADH consumption, upon conversion from Pyr to Lac, is observed when 3PG is added. We used the subtype PK III for this experiment instead of PKM2.

PKM2

**PKM2 beads**	**5 µL**
**LDH (free)**	1 u/µL
**PEP**	1 mM
**ADP**	2 mM
**NADH**	1 mM
**FBP**	1 mM

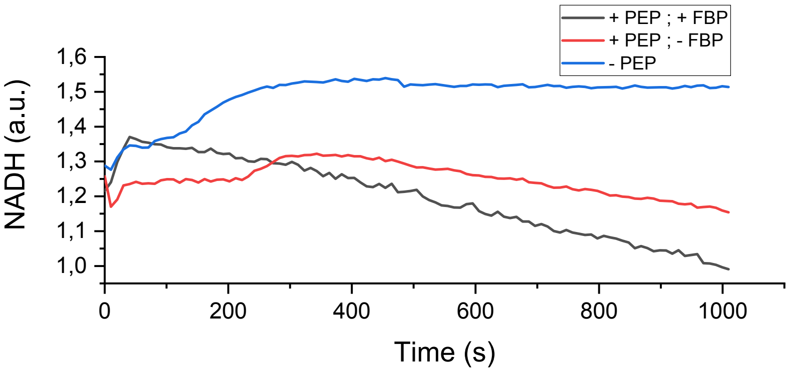

Figure S20. Activity assay of PKM2 beads, with and without an allosteric activator. An excess of free LDH converts Pyr to Lac, the resulting NADH consumption is measured

LDH

**LDH beads**	**5 µL**
**Pyruvate**	1 mM
**NADH**	1 mM

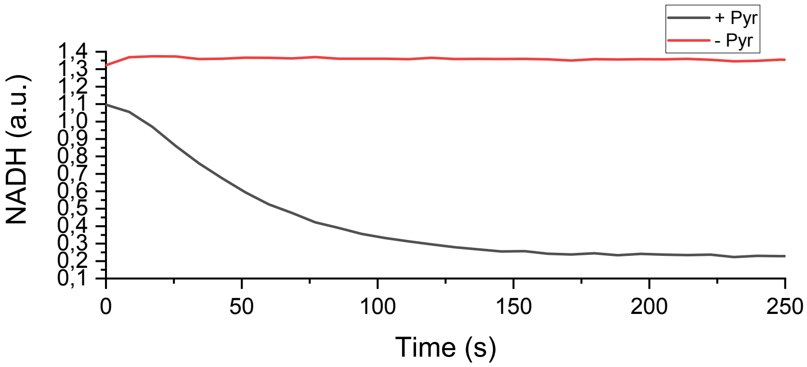

Figure S21. Activity assay of LDH beads. NADH consumption is observed upon conversion of Pyr to Lac.

Nucleotide (U)HPLC

A solution of 100 µL was prepared in a SEPARA ® syringeless filter HPLC vial and left to react for 15 minutes at 21 °C in a Grant-Bio HC-18 Thermoshaker. Subsequently, the reaction was quenched by separating the beads from the solution using a filter, after which 60 µL of the solution was transferred to an HPLC vial with a micro insert and subsequently measured in the Shimadzu Nexera X3 UHPLC with an Inertsil ODS-4 C18 HPLC column 3 µm 150 × 4.6 mm at 40 °C using the following:

We prepared elution solution A as 50 mM aqueous phosphate (pH 6.0) filtered over a 0.22 µm membrane, and solution B as 50% MeOH in water. The elution gradient was as follows: 100% buffer A for 2 min; 0–12.5% linear gradient of buffer B for 8 min; 12.5% buffer B for 2 min; 12.5–40% linear gradient of buffer B for 6 min; 40% buffer B for 2 min; 40–0% linear gradient of buffer B for 3 min; and finally 100% buffer A for 7 min. The flow rate was maintained at 1 ml/min. We match the peaks to the UV signal (detector 254 nm) of ATP (2.9 min), ADP (3.7 min), NAD+ (7.8 min), and NADH (9.4 min) standards.

PGK

**PGK beads**	**5 µL**
**Ald (free)**	1 u/µL
**GAPDH (free)**	1 u/µL
**FBP**	1 mM
**ADP**	2 mM
**NAD$^+$**	2 mM

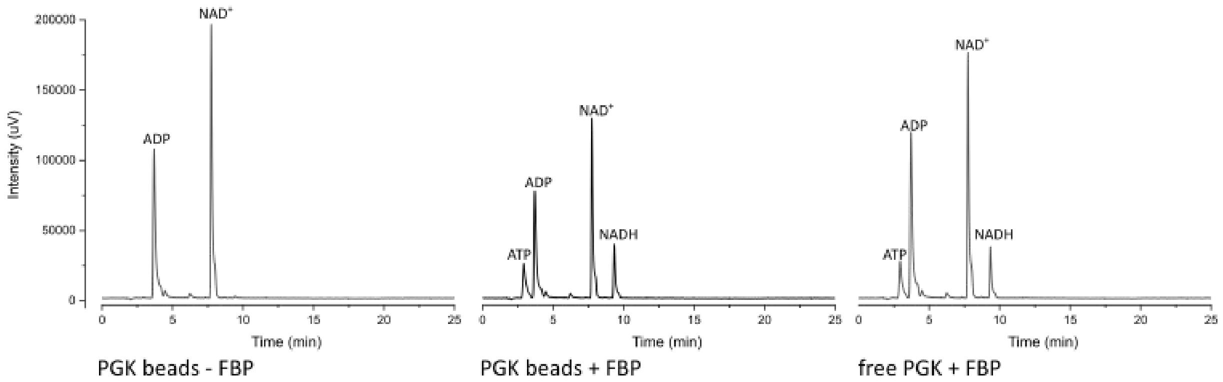

Figure S22. Activity assay of PGK beads. FBP is converted to GAP/DHAP by free aldolase. GAP then reacts with free GAPDH, yielding BPG which is then converted to 3PG by the PGK beads. The consumption of NAD+ and ADP shows that the PGK beads are active.

5. Preparation of stock solutions for analytical standards. We prepared 100 or 125 mM stock solutions of all reagents and analytical standards in Milli-Q. For acidic molecules such as ATP, ADP and NAD^+ we adjusted the pH to 7 through titration with 2.5 M KOH. The glutamic acid standard was kept as a 1 M stock solution and its ^{15}N isotopologue at 25 mM.

All analytical standards were then subjected to quantitative NMR analysis to correct for errors in the preparation of the stock solutions. For all molecules containing a phosphate moiety, we used ^{31}P NMR with HMPA as analytical standard. The other compounds were quantified by ^{1}H NMR with TMSP as analytical standard.

Sample preparation

A sample of 10 µL 10% hexamethylphosphoramide (HMPA) (11.5 mM), 25 µL analyte solution, and 465 µL D_2O were prepared in an NMR tube. Subsequently, ^{31}P NMR was measured quantitatively using a T1 of 30 s.

A sample of 50 µL of 100 mM trimethylsilyl propanoic acid – d4 (TMSP-d4) (10 mM) solution, 25 µL of analyte solution, and 425 µL D_2O were prepared in an NMR tube. Subsequently, ^{1}H NMR was measured quantitatively using a T1 of 22 s.

Quantification

The integral of the internal standard (I_S) is set to 100. We then divide the measured integrals of our analyte I_A by the number of nuclei of that integral n_A. To obtain the actual concentration of analyte dilute into the sample, this quotient is multiplied by the quotient of the number of nuclei n_S of the standard (i.e. 1 for HMPA and 9 for TMSP) and I_S multiplied with the standard sample concentration $[S]$. When divided by the theoretical sample concentration $[A]_t$, and multiplied with the theoretical stock concentration $[A]_T$ the corrected stock concentration $[A]_C$ is obtained (**Equation S9**). The corrected stock concentrations are detailed in **Table S3**.

$$[A]_C = \frac{[A]_T}{[A]_t}\left(\frac{I_A}{n_A}\cdot\frac{n_S}{I_S}\cdot[S]\right) \quad \text{(S9)}$$

6. Mass spectrometry. Ion mobility-mass spectrometry experiments were performed with a timsToF instrument (Bruker, Germany) equipped with an ESI source. Ions were electrosprayed in negative mode with a source voltage of -3.5 kV, with a Nebulizer of 2.0 Bar, a drying gas flow of 8 $L.min^{-1}$, and a source temperature of 250°C. Typical ion transfer voltages were quadrupole ion energy = -5 eV and collision energy = -8 eV. The mass range scanned by the ToF analyzer was *m/z* 50-1050. TIMS experiments were performed in N_2 using the imeX Custom mode, by scanning ion mobility from 0.35 $V.s.cm^{-2}$ to 1.3 $V.s.cm^{-2}$, with a ramp time set at 550 ms. The accumulation time was set to 100 ms. The Bruker ESI needle was replaced with a 15 cm long fused silica capillary tubing (Postnova Z-FSS-100190).

The TIMS dimension was calibrated linearly using three selected ions from the Agilent ESI LC/MS tuning mix [(301.9981, 0.668 $V.s.cm^{-2}$), (601.9790, 0.879 $V.s.cm^{-2}$), (1033.9881, 1.253 $V.s.cm^{-2}$)]. The MS dimension was calibrated quadratically using the ions [112.9856, 301.9981, 601.9790, 1033.9881].

Ion chromatograms were extracted with a width of ± 0.005 Da, and ion chromatograms for F6P/$^{13}C_6$-F6P and G6P/$^{13}C_6$-G6P were extracted for the mobility ranges 0.655-0.665 and 0.675-0.685 $V.s/cm^2$ respectively. Raw ion intensities were normalized by the total ion current (TIC). To accurately determine the ion abundance of ^{15}N-glutamic acid and NADH, the contributions of ^{13}C isotopes of glutamic acid and NAD were removed according to the following table:

**Ion**	**^{13}C isotope contribution (% intensity of the ^{12}C isotope)**
**Glutamic acid**	5.46 % (+1 amu)
**NAD**	2.45% (+2 amu)

Table S4. Contribution of the ^{13}C isotopes for glutamic acid and NAD, at +1 amu and +2 amu respectively.

Since other compounds have more than 2 amu difference with their isotopologues, the contribution of their ^{13}C isotopes was neglected. The data were then binned with a width of 90 frames (~45 s).

7. Setup of the experimental system. A custom made CSTR (Volume = 100 µL), made of poly(methyl methacrylate) was charged with the required volume of each enzyme bead. The inlet and outlet of the reactor were sealed with Whatman Nuclepore polycarbonate membranes (10 µm pore size, cat. number 10418406) to prevent outflow of enzyme beads. We used LABM8 Syringe Pumps and HSW Plastipak 3-part syringes to dose solutions to the CSTR, with all flowrates programmed in gcode via the LABM8 software. The flowrates, bead compositions, and syringe solutions for each experiment can be found in **Tables S5-9**.

Absorbance in flow experiments was continuously measured at the reactor output with a custom 3D printed made flow cell, kindly provided to us by LABM8, connected to an AvaLight 355 nm LED lamp. Absorbance between 340 and 360 nm was detected using an AvaSpec-2048 with 100 ms integration time and averaging for 8 scans. Using a calibration curve, the absorbance data was converted to NADH concentration. UV absorbance data are then exploited to establish a relation between NADH concentration and ion intensities (see **Section 10** and **Figure S30**).

We connected the outflow of the flowcell to a ¼-28 Male x ¼-28 Female check valve inlet (CVI) to eliminate backflow from the dilution line to the CSTR. A Harvard PhD Ultra syringe pump was used to dispense the dilution line, containing all analytical standards, with a flow rate of 87.5 $\mu L.min^{-1}$. The reactor and dilution flow were mixed with a Y- junction, the outflow led to another Y-junction leading either to timsToF instrument or to a manually tuned backpressure regulator (Restek RT-25020; set to ~2 bar) connected to the waste container. A scheme of the entire setup is shown in **Figure S23**.

The first steady state is programmed for at least 2 hours. This equilibration time is planned to allow the whole system to reach a steady pressure.

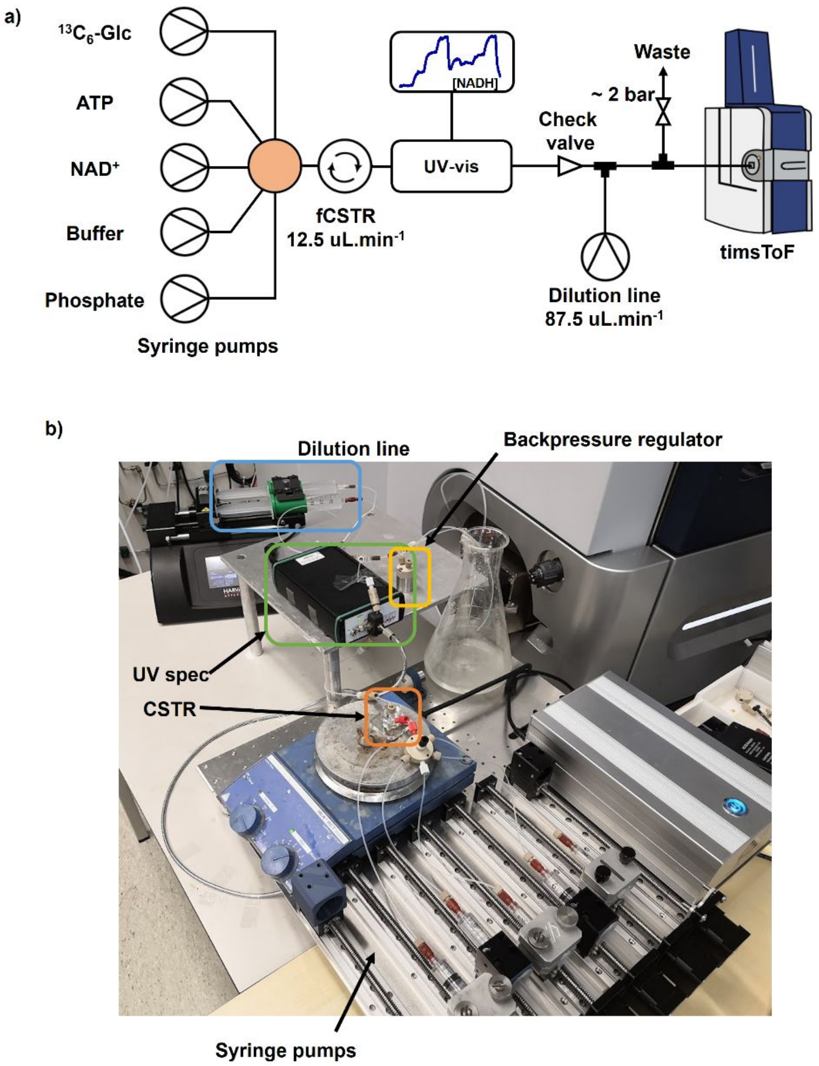

Figure S23. a) Schematic representation of the flow setup. Five syringe pumps containing solutions of $^{13}C_6$-glucose, ATP, NAD^+, buffer and phosphate continuously flow into the continuous stirred tank reactor (CSTR) with a total flow of 12.5 uL.min^{-1}, thereby fixing the residence time inside the CSTR to 8 min (for experiments shown in **Figure 2**, **3** and **S8**). Flowrates are configured with LABM8 syringe pumps. The output flow of the CSTR enters an inline UV spectrometer (Avantes Duo) for absorbance measurement at fixed wavelength. The total flow is diluted with water, using Harvard PhD Ultra syringe pumps, and subsequently split towards the mass spectrometer and a waste line. A manually tuned backpressure regulator diverts flow towards the waste line when the total pressure in the system exceeds ~ 2 bar. **b)** Picture of the flow setup.

8. Compound quantification using isotopologues. To link the ion intensities detected in the mass spectrometer to the concentrations of the respective metabolites in solution, we compared the relative ion intensities of metabolites of unknown concentrations to isotopologues of known concentrations. Under the assumption that isotopologues share the same ionization efficiency, they will be affected in the same way by changes in flowrates, matrix effects or spraying conditions. Therefore, the relative intensities of isotopologues in the mass spectrum directly relate to their relative concentrations in solution.

$^{13}C_6$-labelled hexoses were used as substrates in the CSTR, thereby generating all ^{13}C-labelled metabolites in the cascade of enzymatic reactions. We added known concentrations of non-labelled metabolites in the dilution line. However, flow instabilities in the system may affect the relative concentrations of labelled and non-labelled metabolites. Glutamic acid was thus added to the dilution line, and ^{15}N-labelled glutamic acid was added to the reaction buffer with known concentrations. Glutamic acid should not play a role in the enzymatic cascade taking place in the CSTR. Therefore, the evolution of the relative intensities of the glutamic acid and ^{15}N-labelled glutamic

acid enables the measurement of the flow ratios f between the output flow of the CSTR and the dilution line during the experiment ($f = f_{CSTR} / f_{Dilution}$) (**Figure S24**).[15]

$$f = \frac{I_{15-Glu}}{I_{Glu}} \frac{C_{Glu}}{C_{15-Glu}} \tag{S10}$$

Based on the known concentrations of the metabolites added to the dilution line, and on the flow ratios, the unknown concentrations of ^{13}C-isotopically labelled compounds produced in the CSTR can be determined using **Equation S11**, for instance for the $^{13}C_6$-labelled glucose:

$$C_{13-Glc} = \frac{I_{13-Glc}}{I_{Glc}} \frac{C_{Glc}}{f} \tag{S11}$$

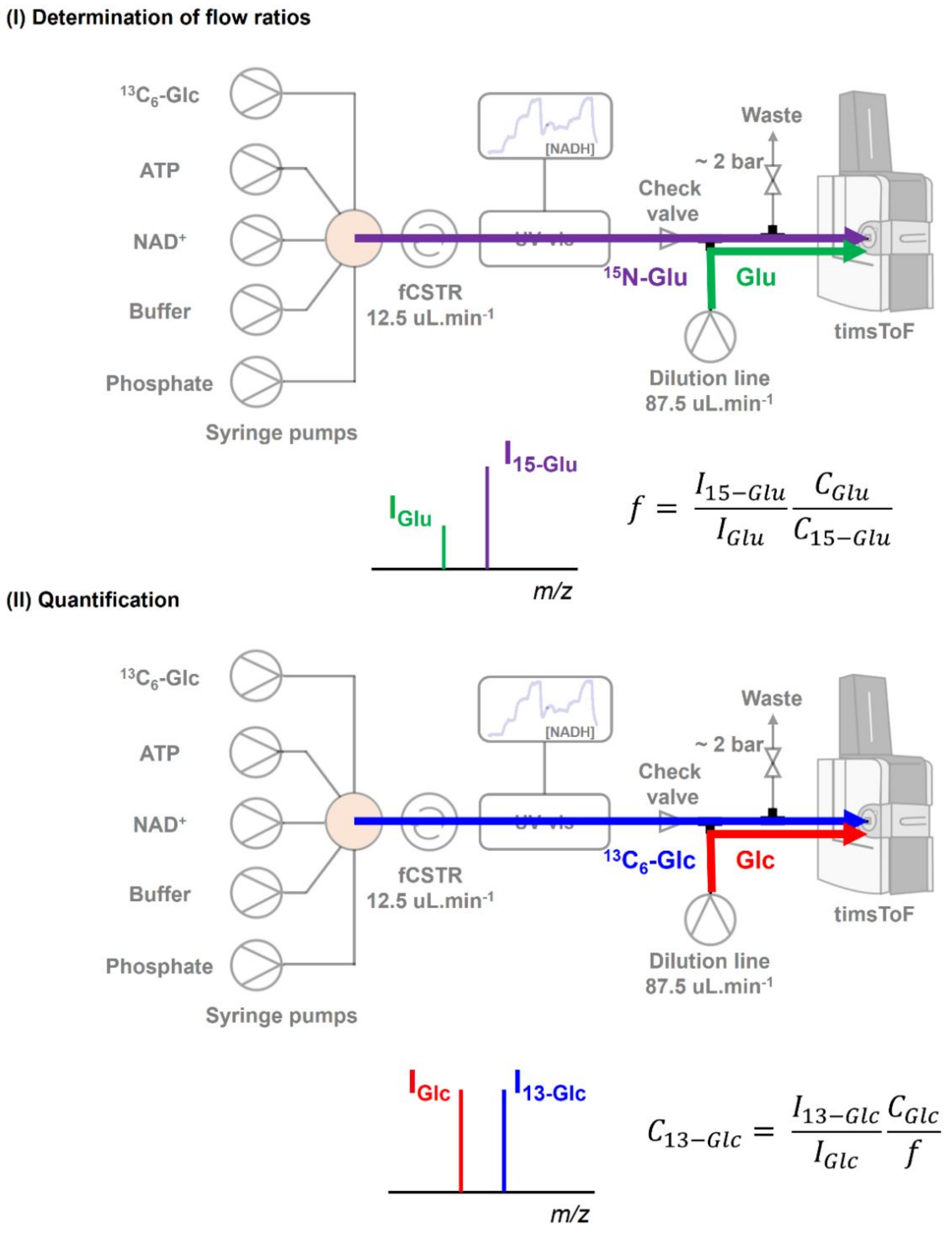

Figure S24. Illustration of the quantification approach. **(I)** The ratio between the output flow from the CSTR and the dilution line are determined from the relative intensities of glutamic acid and ^{15}N-glutamic acid that were added with known concentrations to the dilution line and reaction buffer, respectively. **(II)** ^{13}C isotopically labelled metabolites

flow out of the CSTR, with unknown concentrations. Non isotopically labelled metabolites were added to the dilution line with known metabolites. The unknown concentration of the ^{13}C labelled metabolites flowing out of the CSTR can be determined from the relative intensities of the unlabelled/labelled ions, and the flow ratios. Adapted from reference[15].

9. Correction for in-flight fragmentation. The ESI-MS analysis of individual metabolite standards revealed that some compounds undergo in-flight fragmentation either in the ionization source or in the ion optics of the mass spectrometer. Fragmentation reactions are problematic for our quantification approach when the fragments appear at the same *m/z* as metabolites of interest. For instance, the ESI-MS analysis of a standard of fructose 1,6-biphosphate (FBP) shows the presence of fructose 6-phosphate (F6P) in the mass spectrum, characterized by the *m/z* 259.02 and inverse mobility $1/K_0$ 0.667 V.s/cm.

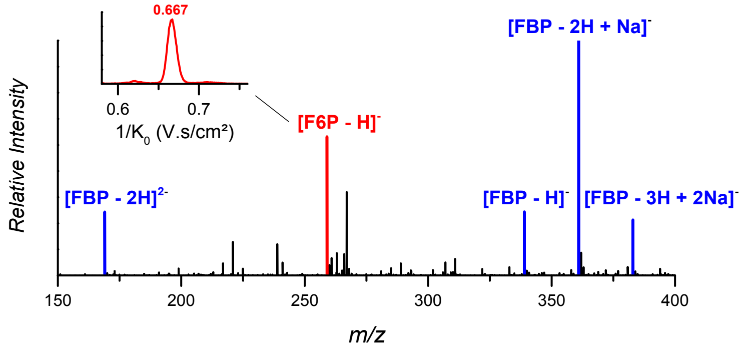

Figure S25. ESI(-)-MS analysis of a 1 mM standard of fructose 1,6-biphosphate (FBP). FBP is detected as deprotonated ions, both singly and doubly charged, and as deprotonated sodium adducts. Fructose 6-phosphate is also detected, and originates from in-flight fragmentation in the mass spectrometer.

The contribution of the FBP fragmentation to the ion intensity of F6P could lead to overestimation of the F6P concentration in our experiments. Assuming that (*i*) isotopologues fragment with similar intensities, and that (*ii*) the fragmentation rate remains constant throughout the experiments, the analysis of metabolites under the same instrumental conditions as our experiments provides estimates of the fragmentation ratio for each metabolite. With this fragmentation rate, the contribution of in-flight fragmentation can be subtracted.

We identified that FBP fragments to F6P, as described above. The analysis of a sample of FBP showed the presence of F6P fragments, however the fragmentation ratio towards F6P is not easily determined from the analysis of a FBP standard. Indeed, by ESI(-)-MS analysis of the standard, FBP ionizes as $[FBP – H]^-$, as sodium adducts $[FBP – 2H + Na]^-$, $[FBP – 3H + 2Na]^-$, or as doubly charged ions $[FBP – 2 H]^{2-}$ while mainly the deprotonated ion is observed in our flow experiments. We determined the fragmentation ratio of FBP to F6P during a trial experiment where only the bottom part of the glycolysis was monitored, starting from FBP. In this experiment, that involved the enzymes Ald, TPI, GAPDH, 3PGK, PGI, Eno, PK and LDH, the detected F6P originates from in-flight fragmentation. By averaging the ion intensity of F6P and FBP on the whole experiment, we estimate that the fragmentation ratio corresponds to 7.1% of the intensity of the deprotonated $[FBP – H]^-$ ion.

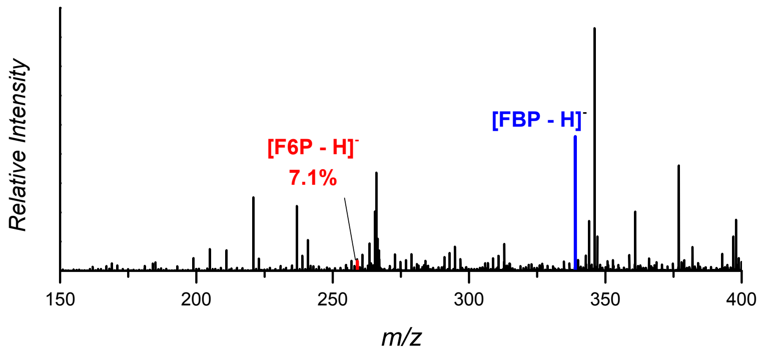

Figure S26. Detection of fructose 6-phosphate during a trial monitoring of the bottom part of the glycolysis, starting from fructose 1,6-biphosphate.

We also identified that glucose fragments to an isomer of lactate, thereby making the fragment ion and the lactate generated by the ERN undistinguishable. The loss of 90 amu is commonly observed in ESI(-)-MS analyses of saccharides.[16, 17] The ESI(-)-MS analysis of a standard of glucose shows that it mostly ionizes as a deprotonated ion $[Glc – H]^-$, similar to our experiments. The ESI(-)-MS analysis of a glucose standard provides an estimate of the fragmentation ratio of glucose into lactate, or an isomer of lactate. As shown in **Figure S27**, we estimate that the fragmentation ratio corresponds to 3.8% of the intensity of the deprotonated $[Glc – H]^-$ ion.

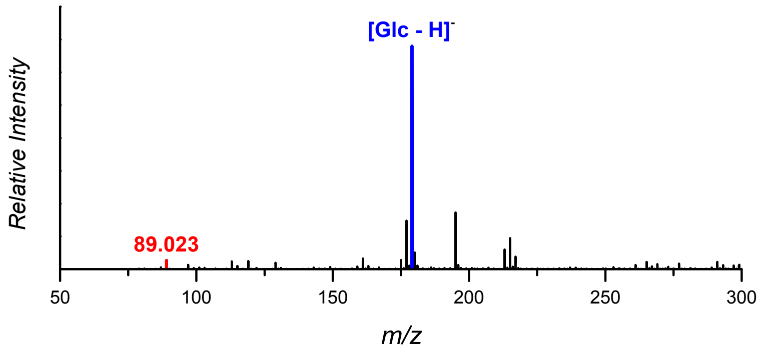

Figure S27. ESI(-)-MS analysis of a 1 mM standard of glucose (Glc). Glucose is mainly detected as deprotonated ion $[Glc – H]^-$, along with ions detected at the mass of deprotonated lactate, or an isomer of lactate.

Similarly, we observed that fructose fragments to lactate, or an isomer of lactate, with a fragmentation ratio of 5.8 % (**Figure S28**).

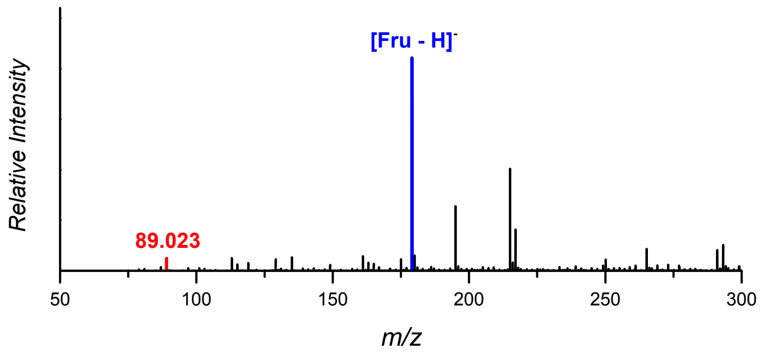

Figure S28. ESI(-)-MS analysis of a 1 mM standard of fructose (Fru). Fructose is mainly detected as deprotonated ion $[Fru – H]^-$, along with ions detected at the mass of deprotonated lactate, or an isomer of lactate.

Finally, we observed ions corresponding to dihydroxyacetone phosphate (DHAP) or glyceraldehyde phosphate (GAP) during the analysis of standards of glucose 6-phosphate (G6P) and fructose 6-phosphate (F6P). Since both G6P and F6P may fragment to DHAP/GAP with different fragmentation ratios, we did not attempt to quantify DHAP/GAP.

It should be noted that the kinetic model established from our data picked out lactate as a consistent outlier (**Figures 4-5** of the main text and **Figure S9**), which indicates that the assumption of consistent fragmentation rates is not always accurate. Nevertheless, it provides an estimate of the concentration range for each metabolite.

10. NADH quantification. The concentration of NADH can be determined based on an absorbance measurement at a fixed wavelength (see section 7). By measuring the absorbance of NADH inflows with known concentrations, the absorbance data can be converted to NADH concentrations (**Figure S29**). However, the UV flow cell is connected to the spectrophotometer by an optical cable that is sensitive to movements. Any shock on the bench may invalidate the calibration during the course of a measurement.

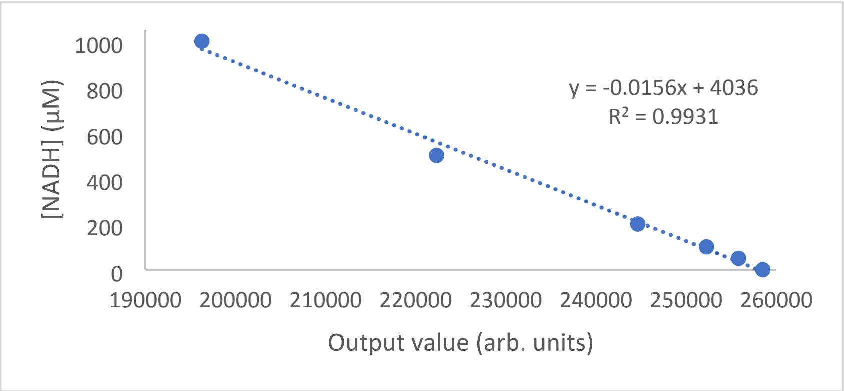

Figure S29. Calibration of the UV flow cell by measuring the absorbance of NADH inflows with known concentrations.

To afford a more reliable readout, we established a linear relationship between NADH concentrations in solution, determined by the UV cell, and ion intensities during one of our ERN experiments. To account for the delay between the UV flow cell and ESI-MS readout, we added a time offset to the UV flow cell readout. As shown in **Figure S30**, a linear correlation can be established between ion intensities and concentrations. This indicates that the NADH concentration range measured in our experiments is within the dynamic range of the mass spectrometer, and that the ionization efficiency of NADH is not affected by matrix effects.

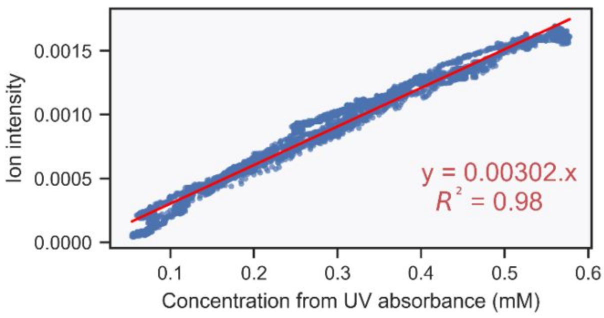

Figure S30. Correlation between ion intensities and concentrations measured by UV absorbance. The data originate from the experiment shown in **Figure 3a** of the main text. The red line corresponds to a linear fit of the data, with the equation and Pearson coefficient indicated in red.

Using this calibration curve, the concentration of NADH can be obtained from ion intensities (**Figure S31a)**. The comparison between NADH concentrations determined either by UV absorbance or ion intensities shows a good correlation (**Figure S31b**).

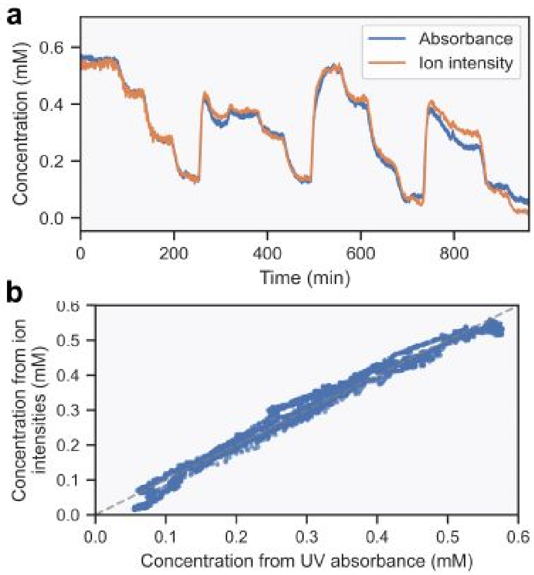

Figure S31. a. NADH concentrations determined either by UV absorbance, or from ion intensities using the relationship from **Figure S30**. **b.** Comparison between NADH concentrations determined by UV absorbance or ion intensities. The grey dashed line represents y = x and corresponds to a perfect match.

The linear relationship drawn in **Figure S30** was used to determine the concentration of NADH from ion intensities in the experiments shown in **Figure 2** and **Figure 3b** of the main text, and in **Figure S8.** As shown by the repeated data in **Figure S8**, the reproducibility of ion intensities between different days ensure an accurate measurement of the NADH concentration. For the experiments shown in **Figure 3a** and **Figure 4**, the UV absorbance readout was used.

OVERVIEW OF THE EXPERIMENTS

Figure 2 (Code SNMS30) :

Time (s)	[$^{13}C_6$ Glucose] (μM) (flowrate)	[ATP] (μM) (flowrate)	[NAD] (μM) (flowrate)	[K_XH_XPO4] (μM) (flowrate)	Buffer flowrate
**0 – 10800**	2000 (375 μL/h)	3000 (180 μL/h)	1500 (90 μL/h)	5000 (18.8 μL/h)	86,2 μL/h
**10800 – 14400**	1000 (187.5 μL/h)	3000 (180 μL/h)	1500 (90 μL/h)	5000 (18.8 μL/h)	273,7 μL/h
**14400 – 18000**	500 (93.8 μL/h)	3000 (180 μL/h)	1500 (90 μL/h)	5000 (18.8 μL/h)	367,5 μL/h
**18000 – 21600**	250 (46.9 μL/h)	3000 (180 μL/h)	1500 (90 μL/h)	5000 (18.8 μL/h)	414,3 μL/h
**21600 – 25200**	2000 (375 μL/h)	1000 (60 μL/h)	1500 (90 μL/h)	5000 (18.8 μL/h)	206,2 μL/h
**25200 – 28800**	1000 (187.5 μL/h)	1000 (60 μL/h)	1500 (90 μL/h)	5000 (18.8 μL/h)	393,7 μL/h
**28800 – 32400**	500 (93.8 μL/h)	1000 (60 μL/h)	1500 (90 μL/h)	5000 (18.8 μL/h)	487,4 μL/h
**32400 – 36000**	250 (46.9 μL/h)	1000 (60 μL/h)	1500 (90 μL/h)	5000 (18.8 μL/h)	534,3 μL/h

Table S5. Steady state concentrations applied to the fCSTR containing hydrogel beads (HK = 2 μL, GPI = 4 μL, G6PDH = 2 μL).

Syringe stocks contained: [$^{13}C_6$ G] = 4 mM; [ATP] = 12.5 mM; [NAD^+] = 12.5 mM, and [K_XH_XPO4] = 200 mM. All solutions, except for the phosphate buffer, were prepared in the following buffer: [ABC] = 50 mM; [KCl] = 20 mM; [$MgCl_2$] = 10 mM. The concentration of ^{15}N-glutamic acid was uneven in different syringes, but this was taken into account during the data analysis. The phosphate buffer (pH 7.8) was prepared in Milli-Q to avoid precipitation in syringe.

Figure 3a (Code SNMS32) :

Time (s)	[$^{13}C_6$ Glucose] (µM) (flowrate)	[ATP] (µM) (flowrate)	[ADP] (µM) (flowrate)	[NAD] (µM) (flowrate)	[K_XH_XPO4] (µM) (flowrate)	Buffer flowrate
**0 - 1800**	2000 (375 µL/h)	1500 (90 µL/h)	1500 (90 µL/h)	1500 (90 µL/h)	5000 (18.75 µL/h)	86.25 µL/h
**1800 – 9000**	2000 (375 µL/h)	2250 (135 µL/h)	750 (45 µL/h)	1500 (90 µL/h)	5000 (18.75 µL/h)	86.25 µL/h
**9000 – 12600**	1000 (187.5 µL/h)	2250 (135 µL/h)	750 (45 µL/h)	1500 (90 µL/h)	5000 (18.75 µL/h)	273.75 µL/h
**12600 – 16200**	500 (93.75 µL/h)	2250 (135 µL/h)	750 (45 µL/h)	1500 (90 µL/h)	5000 (18.75 µL/h)	367.5 µL/h
**16200 – 19800**	250 (46.875 µL/h)	2250 (135 µL/h)	750 (45 µL/h)	1500 (90 µL/h)	5000 (18.75 µL/h)	414.375 µL/h
**19800 – 23400**	2000 (375 µL/h)	750 (45 µL/h)	2250 (135 µL/h)	1500 (90 µL/h)	5000 (18.75 µL/h)	86.25 µL/h
**23400 – 27000**	1000 (187.5 µL/h)	750 (45 µL/h)	2250 (135 µL/h)	1500 (90 µL/h)	5000 (18.75 µL/h)	273.75 µL/h
**27000 – 30600**	500 (93.75 µL/h)	750 (45 µL/h)	2250 (135 µL/h)	1500 (90 µL/h)	5000 (18.75 µL/h)	367.5 µL/h
**34200 – 37800**	250 (46.875 µL/h)	750 (45 µL/h)	2250 (135 µL/h)	1500 (90 µL/h)	5000 (18.75 µL/h)	414.375 µL/h
**37800 – 41400**	2000 (375 µL/h)	2250 (135 µL/h)	750 (45 µL/h)	1000 (60 µL/h)	5000 (18.75 µL/h)	116.25 µL/h
**41400 – 45000**	1000 (187.5 µL/h)	2250 (135 µL/h)	750 (45 µL/h)	1000 (60 µL/h)	5000 (18.75 µL/h)	303.75 µL/h
**45000 – 48600**	500 (93.75 µL/h)	2250 (135 µL/h)	750 (45 µL/h)	1000 (60 µL/h)	5000 (18.75 µL/h)	397.5 µL/h
**48600 – 52200**	250 (46.875 µL/h)	2250 (135 µL/h)	750 (45 µL/h)	1000 (60 µL/h)	5000 (18.75 µL/h)	444.375 µL/h
**52200 – 55800**	2000 (375 µL/h)	750 (45 µL/h)	2250 (135 µL/h)	1000 (60 µL/h)	5000 (18.75 µL/h)	116.25 µL/h
**55800 – 59400**	1000 (187.5 µL/h)	750 (45 µL/h)	2250 (135 µL/h)	1000 (60 µL/h)	5000 (18.75 µL/h)	303.75 µL/h
**59400 – 63000**	500 (93.75 µL/h)	750 (45 µL/h)	2250 (135 µL/h)	1000 (60 µL/h)	5000 (18.75 µL/h)	397.5 µL/h
**63000 – 66600**	250 (46.875 µL/h)	750 (45 µL/h)	2250 (135 µL/h)	1000 (60 µL/h)	5000 (18.75 µL/h)	444.375 µL/h

Table S6. Steady state concentrations applied to the fCSTR containing hydrogel beads (HK = 2.4 µL, GPI = 4.8 µL, PFK = 4.8 µL, G6PDH = 2 µL, Ald = 12 µL, GAPDH = 4.8 µL, TPI = 4.8 µL, PGK = 2.4 µL, PGI = 3.6 µL, Eno = 2.4 µL, PKM2 = 9 µL, LDH = 2.4 µL).

Syringe stocks contained: [$^{13}C_6$ G] = 4 mM; [ATP] = 12.5 mM; [ADP] = 12.5 mM; [NAD^+] = 12.5 mM, and [K_XH_XPO4] = 200 mM. All solutions, except for the phosphate buffer, were prepared in the following buffer: [ABC] = 50 mM; [KCl] = 20 mM; [$MgCl_2$] = 10 mM. The phosphate buffer (pH 7.8) was prepared in Milli-Q to avoid precipitation in syringe. The concentration of ^{15}N-glutamic acid was uneven in different syringes, but this was taken into account during the data analysis.

Note that the first steady state is still in the equilibration phase of the reactor, but uses equal flows of ATP, ADP and NAD^+ to ensure that all syringe pumps flow homogeneously.

Figure 3b (Code SNMS34) :

Time (s)	[$^{13}C_6$ Fructose] (µM) (flowrate)	[ATP] (µM) (flowrate)	[ADP] (µM) (flowrate)	[NAD] (µM) (flowrate)	[K_xH_xPO4] (µM) (flowrate)	Buffer flowrate
**0 - 1800**	4500 (281.25 µL/h)	1500 (90 µL/h)	1500 (90 µL/h)	1500 (90 µL/h)	5000 (18.75 µL/h)	180 µL/h
**1800 – 9000**	4500 (281.25 µL/h)	2250 (135 µL/h)	750 (45 µL/h)	1500 (90 µL/h)	5000 (18.75 µL/h)	180 µL/h
**9000 – 12600**	3000 (187.5 µL/h)	2250 (135 µL/h)	750 (45 µL/h)	1500 (90 µL/h)	5000 (18.75 µL/h)	273.75 µL/h
**12600 – 16200**	1500 (93.75 µL/h)	2250 (135 µL/h)	750 (45 µL/h)	1500 (90 µL/h)	5000 (18.75 µL/h)	367.5 µL/h
**16200 – 19800**	750 (46.875 µL/h)	2250 (135 µL/h)	750 (45 µL/h)	1500 (90 µL/h)	5000 (18.75 µL/h)	414.375 µL/h
**19800 – 23400**	4500 (281.25 µL/h)	750 (45 µL/h)	2250 (135 µL/h)	1500 (90 µL/h)	5000 (18.75 µL/h)	86.25 µL/h
**23400 – 27000**	3000 (187.5 µL/h)	750 (45 µL/h)	2250 (135 µL/h)	1500 (90 µL/h)	5000 (18.75 µL/h)	273.75 µL/h
**27000 – 30600**	1500 (93.75 µL/h)	750 (45 µL/h)	2250 (135 µL/h)	1500 (90 µL/h)	5000 (18.75 µL/h)	367.5 µL/h
**34200 – 37800**	750 (46.875 µL/h)	750 (45 µL/h)	2250 (135 µL/h)	1500 (90 µL/h)	5000 (18.75 µL/h)	414.375 µL/h
**37800 – 41400**	4500 (281.25 µL/h)	2250 (135 µL/h)	750 (45 µL/h)	1000 (60 µL/h)	5000 (18.75 µL/h)	116.25 µL/h
**41400 – 45000**	3000 (187.5 µL/h)	2250 (135 µL/h)	750 (45 µL/h)	1000 (60 µL/h)	5000 (18.75 µL/h)	303.75 µL/h
**45000 – 48600**	1500 (93.75 µL/h)	2250 (135 µL/h)	750 (45 µL/h)	1000 (60 µL/h)	5000 (18.75 µL/h)	397.5 µL/h
**48600 – 52200**	750 (46.875 µL/h)	2250 (135 µL/h)	750 (45 µL/h)	1000 (60 µL/h)	5000 (18.75 µL/h)	444.375 µL/h
**52200 – 55800**	4500 (281.25 µL/h)	750 (45 µL/h)	2250 (135 µL/h)	1000 (60 µL/h)	5000 (18.75 µL/h)	116.25 µL/h
**55800 – 59400**	3000 (187.5 µL/h)	750 (45 µL/h)	2250 (135 µL/h)	1000 (60 µL/h)	5000 (18.75 µL/h)	303.75 µL/h
**59400 – 63000**	1500 (93.75 µL/h)	750 (45 µL/h)	2250 (135 µL/h)	1000 (60 µL/h)	5000 (18.75 µL/h)	397.5 µL/h
**63000 – 66600**	750 (46.875 µL/h)	750 (45 µL/h)	2250 (135 µL/h)	1000 (60 µL/h)	5000 (18.75 µL/h)	444.375 µL/h

Table S7. Steady state concentrations applied to the fCSTR containing hydrogel beads (HK = 2 µL, GPI = 4 µL, PFK = 4 µL, G6PDH = 2 µL, Ald = 10 µL, GAPDH = 4 µL, TPI = 4 µL, PGK = 2 µL, PGI = 3 µL, Eno = 2 µL, PKM2 = 7.5 µL, LDH = 2 µL).

Syringe stocks contained: [$^{13}C_6$ F] = 12 mM; [ATP] = 12.5 mM; [ADP] = 12.5 mM; [NAD^+] = 12.5 mM, and [K_xH_xPO4] = 200 mM. All solutions, except for the phosphate buffer, were prepared in the following buffer: [ABC] = 50 mM; [KCl] = 20 mM; [$MgCl_2$] = 10 mM; [^{15}N-glutamic acid] = 0.5 mM. The phosphate buffer (pH 7.8) was prepared in Milli-Q to avoid precipitation in syringe.

Note that the first steady state is still in the equilibration phase of the reactor, but uses equal flows of ATP, ADP and NAD^+ to ensure that all syringe pumps flow homogeneously.

Figure 4 (Code SNMS37) :

Time (s)	[$^{13}C_6$ Glucose] (µM) (flowrate)	[ATP] (µM) (flowrate)	[ADP] (µM) (flowrate)	[NAD] (µM) (flowrate)	[K_xH_xPO4] (µM) (flowrate)	Buffer flowrate
**0 - 9000**	1000.0 (50.0 µL/h)	4116.66 (95.0 µL/h)	1950.0 (65.0 µL/h)	2700.0 (60.0 µL/h)	5000 (7.5 µL/h)	22.5 µL/h
**9000 - 9900**	400.0 (20.0 µL/h)	7150.0 (165.0 µL/h)	1950.0 (65.0 µL/h)	1125.0 (25.0 µL/h)	5000 (7.5 µL/h)	17.5 µL/h
**9900 - 10800**	2900.0 (145.0 µL/h)	1733.33 (40 µL/h)	1950.0 (65.0 µL/h)	1125.0 (25.0 µL/h)	5000 (7.5 µL/h)	17.5 µL/h
**10800 - 11700**	2900.0 (145.0 µL/h)	2816.66 (65.0 µL/h)	750.0 (25.0 µL/h)	1125.0 (25.0 µL/h)	5000 (7.5 µL/h)	32.5 µL/h
**11700 - 12600**	2600.0 (130.0 µL/h)	3900.0 (90.0 µL/h)	750.0 (25.0 µL/h)	1125.0 (25.0 µL/h)	5000 (7.5 µL/h)	22.5 µL/h
**12600 - 13500**	500.0 (25.0 µL/h)	5633.33 (130 µL/h)	2700.0 (90.0 µL/h)	1125.0 (25.0 µL/h)	5000 (7.5 µL/h)	22.5 µL/h
**13500 - 14400**	500.0 (25.0 µL/h)	3900.0 (90.0 µL/h)	3900.0 (130.0 µL/h)	1125.0 (25.0 µL/h)	5000 (7.5 µL/h)	22.5 µL/h
**14400 - 15300**	1500.0 (75.0 µL/h)	1083.33 (25 µL/h)	1200.0 (40.0 µL/h)	5850.0 (130.0 µL/h)	5000 (7.5 µL/h)	22.5 µL/h
**15300 - 16200**	500.0 (25.0 µL/h)	1083.33 (25 µL/h)	2700.0 (90.0 µL/h)	5850.0 (130.0 µL/h)	5000 (7.5 µL/h)	22.5 µL/h
**16200 - 17100**	500.0 (25.0 µL/h)	1083.33 (25 µL/h)	2700.0 (90.0 µL/h)	5850.0 (130.0 µL/h)	5000 (7.5 µL/h)	22.5 µL/h
**17100 - 18000**	500.0 (25.0 µL/h)	3033.33 (70 µL/h)	3150.0 (105.0 µL/h)	3150.0 (70.0 µL/h)	5000 (7.5 µL/h)	22.5 µL/h
**18000 - 18900**	2100.0 (105.0 µL/h)	2600.0 (60.0 µL/h)	750.0 (25.0 µL/h)	3600.0 (80.0 µL/h)	5000 (7.5 µL/h)	22.5 µL/h
**18900 - 19800**	1800.0 (90.0 µL/h)	3900.0 (90.0 µL/h)	1800.0 (60.0 µL/h)	1125.0 (25.0 µL/h)	5000 (7.5 µL/h)	27.5 µL/h
**19800 - 20700**	1200.0 (100.0 µL/h)	3770.0 (145.0 µL/h)	1080.0 (60.0 µL/h)	4455.0 (165.0 µL/h)	5000 (12.5 µL/h)	17.5 µL/h
**20700 - 21600**	300.0 (25.0 µL/h)	3770.0 (145.0 µL/h)	1980.0 (110.0 µL/h)	4455.0 (165.0 µL/h)	5000 (12.5 µL/h)	42.5 µL/h
**21600 - 22500**	300.0 (25.0 µL/h)	3770.0 (145.0 µL/h)	450.0 (25.0 µL/h)	2160.0 (80.0 µL/h)	5000 (12.5 µL/h)	212.5 µL/h
**22500 - 23400**	1080.0 (90.0 µL/h)	1040.0 (40.0 µL/h)	2340.0 (130.0 µL/h)	3510.0 (130.0 µL/h)	5000 (12.5 µL/h)	97.5 µL/h
**23400 - 24300**	1080.0 (90.0 µL/h)	1040.0 (40.0 µL/h)	450.0 (25.0 µL/h)	3510.0 (130.0 µL/h)	5000 (12.5 µL/h)	202.5 µL/h
**24300 - 25200**	1080.0 (90.0 µL/h)	2860.0 (110.0 µL/h)	450.0 (25.0 µL/h)	3510.0 (130.0 µL/h)	5000 (12.5 µL/h)	132.5 µL/h
**25200 - 26100**	1080.0 (90.0 µL/h)	650.0 (25.0 µL/h)	450.0 (25.0 µL/h)	2835.0 (105.0 µL/h)	5000 (12.5 µL/h)	242.5 µL/h
**26100 - 27000**	1260.0 (105.0 µL/h)	3120.0 (120.0 µL/h)	450.0 (25.0 µL/h)	2835.0 (105.0 µL/h)	5000 (12.5 µL/h)	132.5 µL/h
**27000 - 27900**	300.0 (25.0 µL/h)	5720.0 (220.0 µL/h)	450.0 (25.0 µL/h)	4050.0 (150.0 µL/h)	5000 (12.5 µL/h)	67.5 µL/h
**27900 - 28800**	300.0 (25.0 µL/h)	5720.0 (220.0 µL/h)	450.0 (25.0 µL/h)	4050.0 (150.0 µL/h)	5000 (12.5 µL/h)	67.5 µL/h
**28800 - 29700**	1800.0 (150.0 µL/h)	3900.0 (150.0 µL/h)	450.0 (25.0 µL/h)	2700.0 (100.0 µL/h)	5000 (12.5 µL/h)	62.5 µL/h
**29700 - 30600**	300.0 (25.0 µL/h)	3900.0 (150.0 µL/h)	3600.0 (200.0 µL/h)	2700.0 (100.0 µL/h)	5000 (12.5 µL/h)	12.5 µL/h

**30600 - 31500**	300.0 (25.0 µL/h)	3120.0 (120.0 µL/h)	3240.0 (180.0 µL/h)	3780.0 (140.0 µL/h)	5000 (12.5 µL/h)	22.5 µL/h
**31500 - 32400**	1080.0 (90.0 µL/h)	4680.0 (180.0 µL/h)	2520.0 (140.0 µL/h)	1350.0 (50.0 µL/h)	5000 (12.5 µL/h)	27.5 µL/h
**32400 - 33300**	1440.0 (120.0 µL/h)	3900.0 (150.0 µL/h)	2700.0 (150.0 µL/h)	675.0 (25.0 µL/h)	5000 (12.5 µL/h)	42.5 µL/h
**33300 - 33900**	1114.28 (130 µL/h)	4457.14 (240 µL/h)	2121.43 (165.0 µL/h)	2507.0 (130.0 µL/h)	5000 (17.5 µL/h)	17.5 µL/h
**33900 - 34500**	1200.0 (140.0 µL/h)	4457.14 (240 µL/h)	1414.29 (110 µL/h)	2700.0 (140.0 µL/h)	5000 (17.5 µL/h)	52.5 µL/h
**34500 - 35100**	1371.43 (160 µL/h)	4457.14 (240 µL/h)	1285.71 (100.0 µL/h)	3086.0 (160.01 µL/h)	5000 (17.5 µL/h)	22.5 µL/h
**35100 - 35700**	1028.57 (120.0 µL/h)	464.28 (25 µL/h)	964.28 (75 µL/h)	482.0 (24.99 µL/h)	5000 (17.5 µL/h)	437.5 µL/h
**35700 - 36300**	1028.57 (120.0 µL/h)	4457.14 (240 µL/h)	321.42 (25 µL/h)	482.0 (24.99 µL/h)	5000 (17.5 µL/h)	272.5 µL/h
**36300 - 36900**	1200.0 (140.0 µL/h)	3342.85 (180.0 µL/h)	321.42 (25 µL/h)	482.0 (24.99 µL/h)	5000 (17.5 µL/h)	312.5 µL/h
**36900 - 37500**	1800.0 (210.0 µL/h)	464.28 (25 µL/h)	2314.28 (180 µL/h)	4629.0 (240.02 µL/h)	5000 (17.5 µL/h)	27.5 µL/h
**37500 - 38100**	214.28 (25 µL/h)	3900.0 (210.0 µL/h)	1542.85 (120.0 µL/h)	1446.0 (74.98 µL/h)	5000 (17.5 µL/h)	252.5 µL/h
**38100 - 38700**	214.28 (25 µL/h)	3900.0 (210.0 µL/h)	1542.85 (120.0 µL/h)	4050.0 (210.0 µL/h)	5000 (17.5 µL/h)	117.5 µL/h
**38700 - 39300**	214.28 (25 µL/h)	3900.0 (210.0 µL/h)	2700.0 (210.0 µL/h)	482.0 (24.99 µL/h)	5000 (17.5 µL/h)	212.5 µL/h
**39300 - 39900**	2057.14 (240 µL/h)	2228.57 (120.0 µL/h)	3085.71 (240.0 µL/h)	1446.0 (74.98 µL/h)	5000 (17.5 µL/h)	7.5 µL/h
**39900 - 40500**	2571.42 (300 µL/h)	464.28 (25 µL/h)	2571.43 (200.0 µL/h)	1446.0 (74.98 µL/h)	5000 (17.5 µL/h)	82.5 µL/h
**40500 - 41400**	2785.71 (325.0 µL/h)	464.28 (25 µL/h)	321.43 (25.0 µL/h)	482.0 (24.99 µL/h)	5000 (17.5 µL/h)	282.5 µL/h
**41400 - 42300**	3000.0 (100.0 µL/h)	1625.0 (25.0 µL/h)	1125.0 (25.0 µL/h)	1688.0 (25.01 µL/h)	5000 (5 µL/h)	20.0 µL/h
**42300 - 43200**	750.0 (25.0 µL/h)	4875.0 (75.0 µL/h)	2250.0 (50.0 µL/h)	1688.0 (25.01 µL/h)	5000 (5 µL/h)	20.0 µL/h
**43200 - 44100**	1050.0 (35.0 µL/h)	2925.0 (45.0 µL/h)	3150.0 (70.0 µL/h)	1688.0 (25.01 µL/h)	5000 (5 µL/h)	20.0 µL/h
**44100 - 45000**	900.0 (30.0 µL/h)	1950.0 (30.0 µL/h)	2475.0 (55.0 µL/h)	3712.0 (54.99 µL/h)	5000 (5 µL/h)	25.0 µL/h
**45000 - 45900**	1050.0 (35.0 µL/h)	1625.0 (25.0 µL/h)	2475.0 (55.0 µL/h)	3712.0 (54.99 µL/h)	5000 (5 µL/h)	25.0 µL/h
**45900 - 46800**	1950.0 (65.0 µL/h)	1625.0 (25.0 µL/h)	1800.0 (40.0 µL/h)	2700.0 (40.0 µL/h)	5000 (5 µL/h)	25.0 µL/h
**46800 - 47700**	1050.0 (35.0 µL/h)	3575.0 (55.0 µL/h)	2475.0 (55.0 µL/h)	1688.0 (25.01 µL/h)	5000 (5 µL/h)	25.0 µL/h
**47700 - 48600**	3000.0 (100.0 µL/h)	1625.0 (25.0 µL/h)	1125.0 (25.0 µL/h)	1688.0 (25.01 µL/h)	5000 (5 µL/h)	20.0 µL/h
**48600 - 49500**	1800.0 (60.0 µL/h)	1625.0 (25.0 µL/h)	2700.0 (60.0 µL/h)	1688.0 (25.01 µL/h)	5000 (5 µL/h)	25.0 µL/h
**49500 - 50400**	1200.0 (40.0 µL/h)	2600.0 (40.0 µL/h)	1800.0 (40.0 µL/h)	2700.0 (40.0 µL/h)	5000 (5 µL/h)	35.0 µL/h
**50400 - 51300**	750.0 (25.0 µL/h)	2600.0 (40.0 µL/h)	1800.0 (40.0 µL/h)	2700.0 (40.0 µL/h)	5000 (5 µL/h)	50.0 µL/h
**51300 - 54900**	300.0 (10.0 µL/h)	2600.0 (40.0 µL/h)	1800.0 (40.0 µL/h)	2700.0 (40.0 µL/h)	5000 (5 µL/h)	65.0 µL/h

Table S8. Steady state concentrations applied to the fCSTR containing hydrogel beads (HK = 2 µL, GPI = 4 µL, PFK = 4 µL, G6PDH = 2 µL, Ald = 10 µL, GAPDH = 4 µL, TPI = 4 µL, PGK = 2 µL, PGI = 3 µL, Eno = 2 µL, PKM2 = 7.5 µL, LDH = 2 µL).

Syringe stocks contained: [$^{13}C_6$ G] = 6 mM; [ATP] = 13 mM; [ADP] = 9 mM; [NAD^+] = 13.5 mM, and [K_XH_XPO4] = 200 mM. All solutions, except for the phosphate buffer, were prepared in the following buffer: [ABC] = 50 mM; [KCl] = 20 mM; [$MgCl_2$] = 10 mM; [^{15}N-glutamic acid] = 0.5 mM. The phosphate buffer (pH 7.8) was prepared in Milli-Q to avoid precipitation in syringe.

Figure S8 (Code SNMS35) :

Time (s)	[$^{13}C_6$ Fructose] (µM) (flowrate)	[ATP] (µM) (flowrate)	[ADP] (µM) (flowrate)	[NAD] (µM) (flowrate)	[K_XH_XPO4] (µM) (flowrate)	Buffer flowrate
**0 - 1800**	4500 (281.25 µL/h)	1500 (90 µL/h)	1500 (90 µL/h)	1500 (90 µL/h)	5000 (18.75 µL/h)	180 µL/h
**1800 – 9000**	4500 (281.25 µL/h)	2250 (135 µL/h)	750 (45 µL/h)	1500 (90 µL/h)	5000 (18.75 µL/h)	180 µL/h
**9000 – 12600**	3000 (187.5 µL/h)	2250 (135 µL/h)	750 (45 µL/h)	1500 (90 µL/h)	5000 (18.75 µL/h)	273.75 µL/h
**12600 – 16200**	1500 (93.75 µL/h)	2250 (135 µL/h)	750 (45 µL/h)	1500 (90 µL/h)	5000 (18.75 µL/h)	367.5 µL/h
**16200 – 19800**	750 (46.875 µL/h)	2250 (135 µL/h)	750 (45 µL/h)	1500 (90 µL/h)	5000 (18.75 µL/h)	414.375 µL/h
**19800 – 23400**	4500 (281.25 µL/h)	750 (45 µL/h)	2250 (135 µL/h)	1500 (90 µL/h)	5000 (18.75 µL/h)	86.25 µL/h
**23400 – 27000**	3000 (187.5 µL/h)	750 (45 µL/h)	2250 (135 µL/h)	1500 (90 µL/h)	5000 (18.75 µL/h)	273.75 µL/h
**27000 – 30600**	1500 (93.75 µL/h)	750 (45 µL/h)	2250 (135 µL/h)	1500 (90 µL/h)	5000 (18.75 µL/h)	367.5 µL/h
**34200 – 37800**	750 (46.875 µL/h)	750 (45 µL/h)	2250 (135 µL/h)	1500 (90 µL/h)	5000 (18.75 µL/h)	414.375 µL/h
**37800 – 41400**	4500 (281.25 µL/h)	2250 (135 µL/h)	750 (45 µL/h)	1000 (60 µL/h)	5000 (18.75 µL/h)	116.25 µL/h
**41400 – 45000**	3000 (187.5 µL/h)	2250 (135 µL/h)	750 (45 µL/h)	1000 (60 µL/h)	5000 (18.75 µL/h)	303.75 µL/h
**45000 – 48600**	1500 (93.75 µL/h)	2250 (135 µL/h)	750 (45 µL/h)	1000 (60 µL/h)	5000 (18.75 µL/h)	397.5 µL/h
**48600 – 52200**	750 (46.875 µL/h)	2250 (135 µL/h)	750 (45 µL/h)	1000 (60 µL/h)	5000 (18.75 µL/h)	444.375 µL/h
**52200 – 55800**	4500 (281.25 µL/h)	750 (45 µL/h)	2250 (135 µL/h)	1000 (60 µL/h)	5000 (18.75 µL/h)	116.25 µL/h
**55800 – 59400**	3000 (187.5 µL/h)	750 (45 µL/h)	2250 (135 µL/h)	1000 (60 µL/h)	5000 (18.75 µL/h)	303.75 µL/h
**59400 – 63000**	1500 (93.75 µL/h)	750 (45 µL/h)	2250 (135 µL/h)	1000 (60 µL/h)	5000 (18.75 µL/h)	397.5 µL/h
**63000 – 66600**	750 (46.875 µL/h)	750 (45 µL/h)	2250 (135 µL/h)	1000 (60 µL/h)	5000 (18.75 µL/h)	444.375 µL/h

Table S9. Steady state concentrations applied to the fCSTR containing hydrogel beads (HK = 2 µL, GPI = 4 µL, PFK = 4 µL, G6PDH = 2 µL, Ald = 10 µL, GAPDH = 4 µL, TPI = 4 µL, PGK = 2 µL, PGI = 3 µL, Eno = 2 µL, PKM2 = 7.5 µL, LDH = 2 µL).

Syringe stocks contained: [$^{13}C_6$ F] = 12 mM; [ATP] = 12.5 mM; [ADP] = 12.5 mM; [NAD^+] = 12.5 mM, and [K_XH_XPO4] = 200 mM. All solutions, except for the phosphate buffer, were prepared in the following buffer: [ABC] = 50 mM; [KCl] = 20 mM; [$MgCl_2$] = 10 mM; [^{15}N-glutamic acid] = 0.5 mM. The phosphate buffer (pH 7.8) was prepared in Milli-Q to avoid precipitation in syringe.

Note that the first steady state is still in the equilibration phase of the reactor, but uses equal flows of ATP, ADP and NAD^+ to ensure that all syringe pumps flow homogeneously.

SOFTWARE AND DATA AVAILABILITY

The software itself is written in Python 3.8 (python software foundation, Delaware US). Code can be found at Huckgroup Github at http://github.com/huckgroup/OED archived with DOI: 10.5281/zenodo.10411170 (2023). The OED utilizes the AMICI solver which is an ODE compilation package to C++ software that is continuously updated.[18-22] The algorithm performs the bulk of the calculations (solving ODEs) in AMICI, a tool that is thoroughly benchmarked and is widely used.[22] Raw ion intensities and notebooks used to generate the figures can be found at Huckgroup Github at https://github.com/huckgroup/IMS-MS_Glycolysis

REFERENCES

1. A. F. Villaverde, D. Pathirana, F. Frohlich, J. Hasenauer and J. R. Banga, A protocol for dynamic model calibration, *Briefings in Bioinformatics*, 2022, **23**, 1-19.
2. A. F. Villaverde, E. Raimundez, J. Hasenauer and J. R. Banga, Assessment of Prediction Uncertainty Quantification Methods in Systems Biology, *IEEE/ACM Transactions on Computational Biology and Bioinformatics*, 2023, **20**, 1725-1736.
3. A. Raue, C. Kreutz, T. Maiwald, J. Bachmann, M. Schilling, U. Klingmuller and J. Timmer, Structural and practical identifiability analysis of partially observed dynamical models by exploiting the profile likelihood, *Bioinformatics*, 2009, **25**, 1923-1929.
4. A. Sinkoe and J. Hahn, Optimal Experimental Design for Parameter Estimation of an IL-6 Signaling Model, *Processes*, 2017, **5**, 49.
5. H. Cramér, *Mathematical Methods of Statistics*, Princeton University Press, 1999.
6. J. Ruess, F. Parise, A. Milias-Argeitis, M. Khammash and J. Lygeros, Iterative experiment design guides the characterization of a light-inducible gene expression circuit, *Proceedings of the National Academy of Sciences of the United States of America*, 2015, **112**, 8148-8153.
7. P. F. de Aguiar, B. Bourguignon, M. S. Khots, D. L. Massart and R. Phan-Than-Luu, D-optimal designs, *Chemometrics Intellig. Lab. Syst.*, 1995, **30**, 199-210.
8. B. van Sluijs, R. J. M. Maas, A. J. van der Linden, T. F. A. de Greef and W. T. S. Huck, A microfluidic optimal experimental design platform for forward design of cell-free genetic networks, *Nat. Commun.*, 2022, **13**, 3626.
9. B. van Sluijs, T. Zhou, B. Helwig, M. G. Baltussen, F. H. T. Nelissen, H. A. Heus and W. T. S. Huck, Iterative design of training data to control intricate enzymatic reaction networks, *Nat. Commun.*, 2024, **15**, 1602.
10. J. M. Rohwer, A. J. Hanekom and J.-H. S. Hofmeyr, A Universal Rate Equation for Systems Biology, *Experimental standard conditions of enzyme characterizations. Proceedings of the 2nd International Beilstein Workshop*, 2007, 175-188.
11. P. F. Cook and W. W. Cleland, *Enzyme Kinetics and Mechanism*, Taylor & Francis, 1 edn., 2007.
12. M. G. Baltussen, J. van de Wiel, C. L. Fernandez Regueiro, M. Jakstaite and W. T. S. Huck, A Bayesian Approach to Extracting Kinetic Information from Artificial Enzymatic Networks, *Anal. Chem.*, 2022, **94**, 7311-7318.
13. A. Pandi, C. Diehl, A. Yazdizadeh Kharrazi, S. A. Scholz, E. Bobkova, L. Faure, M. Nattermann, D. Adam, N. Chapin, Y. Foroughijabbari, C. Moritz, N. Paczia, N. S. Cortina, J. L. Faulon and T. J. Erb, A versatile active learning workflow for optimization of genetic and metabolic networks, *Nat. Commun.*, 2022, **13**, 3876.
14. A. Gabor, A. F. Villaverde and J. R. Banga, Parameter identifiability analysis and visualization in large-scale kinetic models of biosystems, *BMC Systems Biology*, 2017, **11**, 54.
15. C. Hold, S. Billerbeck and S. Panke, Forward design of a complex enzyme cascade reaction, *Nat. Commun.*, 2016, **7**, 12971.
16. D. Wan, H. Yang, C. Yan, F. Song, Z. Liu and S. Liu, Differentiation of glucose-containing disaccharides isomers by fragmentation of the deprotonated non-covalent dimers using negative electrospray ionization tandem mass spectrometry, *Talanta*, 2013, **115**, 870-875.
17. E. Amoah, D. S. Kulyk, C. S. Callam, C. M. Hadad and A. K. Badu-Tawiah, Mass Spectrometry Approach for Differentiation of Positional Isomers of Saccharides: Toward Direct Analysis of Rare Sugars, *Anal. Chem.*, 2023, **95**, 5635-5642.
18. F. Frohlich, D. Weindl, Y. Schalte, D. Pathirana, L. Paszkowski, G. T. Lines, P. Stapor and J. Hasenauer, AMICI: high-performance sensitivity analysis for large ordinary differential equation models, *Bioinformatics*, 2021, **37**, 3676-3677.
19. P. Lakrisenko, P. Stapor, S. Grein, L. Paszkowski, D. Pathirana, F. Frohlich, G. T. Lines, D. Weindl and J. Hasenauer, Efficient computation of adjoint sensitivities at steady-state in ODE models of biochemical reaction networks, *PLoS Comp. Biol.*, 2023, **19**, e1010783.
20. Y. Schälte, F. Fröhlich, P. J. Jost, J. Vanhoefer, D. Pathirana, P. Stapor, P. Lakrisenko, D. Wang, E. Raimúndez, S. Merkt, L. Schmiester, P. Städter, S. Grein, E. Dudkin, D. Doresic, D. Weindl and J. Hasenauer, pyPESTO: a modular and scalable tool for parameter estimation for dynamic models, *Bioinformatics*, 2023, **39**, btad711.
21. P. Städter, Y. Schälte, L. Schmiester, J. Hasenauer and P. L. Stapor, Benchmarking of numerical integration methods for ODE models of biological systems, *Sci. Rep.*, 2021, **11**, 2696.
22. L. Schmiester, Y. Schalte, F. T. Bergmann, T. Camba, E. Dudkin, J. Egert, F. Frohlich, L. Fuhrmann, A. L. Hauber, S. Kemmer, P. Lakrisenko, C. Loos, S. Merkt, W. Muller, D. Pathirana, E. Raimundez, L. Refisch, M. Rosenblatt, P. L. Stapor, P. Stadter, D. Wang, F. G. Wieland, J. R. Banga, J. Timmer, A. F. Villaverde, S. Sahle, C. Kreutz, J. Hasenauer and D. Weindl, PEtab-Interoperable specification of parameter estimation problems in systems biology, *PLoS Comp. Biol.*, 2021, **17**, e1008646.
